# Oxidative Stress Activated by Sorafenib Alters the Temozolomide Sensitivity of Human Glioma Cells Through Autophagy and JAK2/STAT3-AIF Axis

**DOI:** 10.3389/fcell.2021.660005

**Published:** 2021-06-14

**Authors:** Jianwei Wei, Zhengfeng Wang, Weiwei Wang, Xiaoge Liu, Junhu Wan, Yongjie Yuan, Xueyuan Li, Liwei Ma, Xianzhi Liu

**Affiliations:** ^1^Department of Neurosurgery, The First Affiliated Hospital of Zhengzhou University, Zhengzhou, China; ^2^Department of Pathology, The First Affiliated Hospital of Zhengzhou University, Zhengzhou, China; ^3^Department of Magnetic Resonance Imaging, The First Affiliated Hospital of Zhengzhou University, Zhengzhou, China; ^4^Department of Clinical Laboratory, The First Affiliated Hospital of Zhengzhou University, Zhengzhou, China; ^5^Department of Interventional Neurology, The First Affiliated Hospital of Zhengzhou University, Zhengzhou, China

**Keywords:** JAK2/STAT3, AIF, sorafenib, TMZ, glioma cells

## Abstract

The development of temozolomide (TMZ) resistance in glioma leads to poor patient prognosis. Sorafenib, a novel diaryl urea compound and multikinase inhibitor, has the ability to effectively cross the blood-brain barrier. However, the effect of sorafenib on glioma cells and the molecular mechanism underlying the ability of sorafenib to enhance the antitumor effects of TMZ remain elusive. Here, we found that sorafenib could enhance the cytotoxic effects of TMZ in glioma cells *in vitro* and *in vivo*. Mechanistically, the combination of sorafenib and TMZ induced mitochondrial depolarization and apoptosis inducing factor (AIF) translocation from mitochondria to nuclei, and this process was dependent on STAT3 inhibition. Moreover, the combination of sorafenib and TMZ inhibited JAK2/STAT3 phosphorylation and STAT3 translocation to mitochondria. Inhibition of STAT3 activation promoted the autophagy-associated apoptosis induced by the combination of sorafenib and TMZ. Furthermore, the combined sorafenib and TMZ treatment induced oxidative stress while reactive oxygen species (ROS) clearance reversed the treatment-induced inhibition of JAK2/STAT3. The results indicate that sorafenib enhanced the temozolomide sensitivity of human glioma cells by inducing oxidative stress-mediated autophagy and JAK2/STAT3-AIF axis.

## Introduction

Glioma is a common malignant tumor of the central nervous system (CNS) that accounts for approximately 45% of all intracranial tumors (Li et al., 2020). The current standard treatment for glioma is surgery followed by radiotherapy and chemotherapy (Ganipineni et al., 2018; Collins and Pollack, 2020), and TMZ is often administered as adjuvant chemotherapy (Lan et al., 2020). However, the development of TMZ resistance promotes the survival of glioma cells and leads to poor prognosis of patients. To overcome TMZ resistance, studies have found that metformin or sulfasalazine could enhance the cytotoxicity of TMZ in glioblastoma cells (Ignarro et al., 2016). Mechanistically, DNA repair systems, including DNA mismatch repair (MMR) (Perazzoli et al., 2015) and base excision repair (BER) (Tang et al., 2011), play important roles in the mechanisms of TMZ resistance. In addition, epidermal growth factor receptor (EGFR) (Chong et al., 2015), murine double minute 2 (Mdm2) (Costa et al., 2013) and the PI3K/AKT/mTOR pathway (Liu et al., 2015) are involved in TMZ resistance mechanisms. Reports have concluded that TMZ could kill most of the original tumor cells; however, tumor stem (initiating) cells are considered to naturally resist to radiochemotherapy and represent a primary cause of tumor recurrence after treatment (Miyazaki et al., 2020). Therefore, it is urgent to improve the sensitivity of glioma to TMZ.

Signal transductor and transcriptional activator 3 (STAT3) is involved in transferring signals from the plasma membrane to the nucleus and modulating cell survival and metastasis (Ma et al., 2020; Su et al., 2020b). Activation of STAT3 is often associated with poor prognosis and chemotherapy resistance in cancer, such as glioma (Li et al., 2018; Kim et al., 2020). The newly identified cancer-promoting role of STAT3 in mitochondria further emphasizes the importance of targeting STAT3 (Su et al., 2020a). STAT3 is involved in regulating the activity of electron transport chain (ETC) complexes I, II, and V and binds to the mitochondrial genome, thereby affecting mitochondrial function (Macias et al., 2014). Moreover, STAT3 can localize to the mitochondria and regulate the concentration of ROS (Garama et al., 2015; Luo et al., 2020). When mitochondrial function is impaired, the proapoptotic Bcl-2 proteins BAX and BAK cause programmed cell death through penetration of the outer mitochondrial membrane (OMM) (Hlavac et al., 2019; Hu et al., 2020). Moreover, impaired mitochondrial function activates AIF, which is released from the mitochondria, translocates into the nucleus, and subsequently initiates the caspase cascade and the intrinsic apoptotic pathway (Choudhury et al., 2010). In hepatocellular carcinoma cells, sorafenib inhibits STAT3 activity by dephosphorylating STAT3 and leads to the downregulation of Mcl-1 (Xie et al., 2018). Sorafenib is a novel diaryl urea compound and multikinase inhibitor that specifically reduces the activity of Raf kinase (Hung et al., 2014). In addition, studies have observed a synergistic antitumor effect between sorafenib and conventional chemotherapy drugs in cancer, such as pancreatic cancer (Booth et al., 2020), and in cancer stem cells (Nawara et al., 2020). Moreover, sorafenib has the ability to effectively cross the blood-brain barrier and sorafenib treatment is well-tolerated by patients (Siegelin et al., 2010). Presently, the efficacy of sorafenib and temozolomide in glioma is still controversial. Although a previous report indicated that the combination of sorafenib and temozolomide is feasible and safe and exhibits activity in patients with relapsed GBM (Zustovich et al., 2013), another study reported that sorafenib treatment may not improve the efficacy of radiochemotherapy in GBM (Riedel et al., 2016). In a phase 2 trial, the combination of sorafenib and temozolomide was used as the first-line treatment for patients with glioblastoma multiforme but did not obviously improve the effect of traditional combined therapy. This result may be related to the high early dropout rate in the study (Hainsworth et al., 2010). Therefore, the role and mechanism of combined treatment with sorafenib and TMZ in glioma must be clarified.

First, we investigated the cytotoxic effect of combined treatment with sorafenib and TMZ on human glioma cells. Then, we detected the effects of this combined therapy on apoptosis and mitochondrial function and examined the mechanisms underlying the ability of the combined therapy to promote cell apoptosis via oxidative stress-mediated autophagy and JAK2/STAT3-AIF axis. Finally, we investigated the ability of sorafenib to enhance the antitumor effects of the conventional chemotherapeutic agent TMZ *in vitro*.

## Materials and Methods

### Cell Lines

The human glioma cell lines U251, LN18 and SHG-44 and the rat glioma cell line C6 were purchased from Shanghai Institute of Cell Biology, Chinese Academy of Sciences (Shanghai, China). The cells were cultured in DMEM (Gibco, Carlsbad, CA, United States) supplemented with 10% fetal bovine serum (Invitrogen, Carlsbad, CA, United States) at 37°C in 5% CO^2^.

### Cell Viability Assays

The glioma cell lines U251, LN18, SHG-44 and C6 were seeded in 96-well plates at a density of 5 × 10^4^ cells/well and cultured for 24 h. The cells were then treated with 0.5, 1.0, 2.0, 4.0, 8.0, and 16.0 μM sorafenib for 24 h and 48 h and 3.125, 6.25, 12.5, 25, 50, 100, and 200 μM TMZ for 24 and 48 h. The control was cultured with medium containing an appropriate amount of DMSO. For the combination of sorafenib and TMZ, the doses of sorafenib and TMZ were 2 and 100 μM for 24 h, respectively. After incubation, cellular viability was detected with an MTT assay by adding 20 μl of MTT (3-(4,5-dimethylthiazol-2-yl)-2,5– diphenyltetrazolium bromide; 5 mg/ml in PBS) to each well for 4–6 h. Then, 150 μl of dimethyl sulfoxide (Beijing Chemical Industry Limited Company, China) was added to each well. The absorbance was detected at a wavelength of 570 nm using a Vmax Microplate Reader (Molecular Devices, Sunnyvale, CA, United States).

### Immunofluorescence Confocal Laser Microscopy

U251 (1 × 10^5^ cells/well) and SHG-44 (1 × 10^5^ cells/well) glioma cells were seeded in a 24-well microplate and cultured for 12 h or 24 h. The control was cultured with medium containing an appropriate amount of DMSO. After administering treatment with the indicated sorafenib and TMZ concentrations and time periods, the cells were fixed with 4% paraformaldehyde for 20 min and incubated with 1% Triton X-100 for 10 min. The nonspecific antibody-binding sites on the cells were blocked with 10% goat serum for 30 min. The cells were then incubated with the following primary antibodies: cleaved caspase 3, SQSTM1/p62 (p62), LC3, and Beclin1 (1:100). Then, the cells were incubated with FITC- or Texas Red-conjugated secondary antibodies (1:200) (Santa Cruz Biotechnology, CA, United States) for 30 min, followed by incubation with Hoechst 33342 solution (Sigma-Aldrich, St. Louis, MO) for 2 min at room temperature. Finally, the cells were visualized at 120× magnification with an Olympus FV1000 confocal laser microscope.

### Western Blot Analysis

U251 (4 × 105 cells/well) and SHG-44 (4 × 105 cells/well) cells were seeded into 6-well microplates. The control was cultured with medium containing an appropriate amount of DMSO. After treatment with 2 μM sorafenib and 100 μM TMZ, 120 μl RIPA lysis buffer was added to each well of the 6-well plate. Glioma tissues were cut into tiny pieces, and lysis buffer was added at a ratio of 200 μl RIPA lysis buffer/20 mg tissue. The tissues were ground with liquid nitrogen after freezing and then added to RIPA lysis buffer after full grinding. The liquid was collected and centrifuged at 12,000 g for 5 min to obtain the supernatants. The protein content of the supernatant was determined using a Bio-Rad protein assay kit (Bio-Rad, Hercules, CA, United States). After 10–15% SDS-PAGE and transfer, the PVDF membranes were blocked with 5% skim milk for 60 min at room temperature and then incubated overnight at 4°C with primary antibodies. Anti-Bax, anti-Bcl-2, anti-p62, anti-STAT3, anti-Beclin-1, anti-JAK2, anti-TOM20 and anti-AIF antibody (1:200 dilution) were obtained from Santa Cruz Biotechnology, United States. Anti-β-actin antibody (1:5,000 dilution) were obtained from Shanghai Abways Biotechnology Co., Ltd., China. Anti-p-STAT3, anti-p-JAK2, anti-cytochrome c (Cyt c), and anti-caspase-3 antibody (1:1,000 dilution) were obtained from Abcam (Hong Kong) Ltd., Hong Kong. Anti- Histone-3 antibody and anti-LC3 (1:1,000 dilution) was obtained from Cell Signaling Technology, United States. After incubation with the horseradish peroxidase-conjugated secondary antibody (1:2,000; Thermo Fisher Scientific, Waltham, MA), the blots were washed 3 times with PBST and immunoreactive proteins were visualized using ECL reagents and captured by Syngene Bio Imaging (Synoptics, Cambridge, United Kingdom). Densitometry was performed using Quantity One software (Bio-Rad).

### Apoptosis Analysis by Flow Cytometry

U251 (4 × 10^5^ cells/well) and SHG-44 (4 × 10^5^ cells/well) glioma cells were seeded into 6-well microplates, incubated for 24 h, and treated with the target compounds. Cell apoptosis was assessed by a FITC Annexin V Apoptosis Detection Kit according to the provided protocol (Beyotime Institute of Biotechnology). Cells were centrifuged at 1,000 g for 5 min and washed twice with PBS. Then, the collected cells were gently resuspended in PBS and counted. A total of 8 × 10^4^ resuspended cells were centrifuged at 1,000 g for 5 min. The supernatant was then discarded, and the cells were gently resuspended in 195 μl Annexin V-FITC binding solution. Subsequently, 5 μl Annexin V-FITC was added and the solution was mixed gently. Then, 10 μl propidium iodide staining solution was added and the solution was mixed gently. The cells were incubated for 15 min at room temperature in the dark, placed in an ice bath, resuspended 2–3 times during incubation to improve staining, and then analyzed by flow cytometry (Becton-Dickinson, Franklin Lakes, NJ, United States).

### RNA Interference

Knockdown of the AIF gene was performed by siRNA in U251 cells. AIF siRNA and negative control siRNA were purchased from GenePharma (Shanghai, China). The siRNA sequences were as follows: human AIF, 5′-GCAGUGGCAAGUUACUUAUTT-3′, and control siRNA (Scramble), 5′-UUCUCCGAACGUGUCACGUTT-3′ (Zhao et al., 2016). U251 cells were transfected using Lipofectamine 2000 (Invitrogen) according to the manufacturer’s instructions.

### Mitochondrial Membrane Potential (MMP, ΔΨm) Assay

U251 (4 × 10^5^ cells/well) cells were seeded in 6-well plates and then treated with 2 μM sorafenib and 100 μM TMZ at the indicated times. The control was cultured with medium containing an appropriate amount of DMSO. The cells were collected and gently resuspended in cell culture medium. Then, the corresponding volume of JC-1 dyeing solution and mix were added and the cells were incubated at 37°C for 20 min. Subsequently, the cells were centrifuged at 600 g at 4°C for 3 min. Then, the supernatant was discarded, 1 × JC-1 staining buffer (Beyotime Biotech, Nanjing, China) was added to resuspend the cells, and the cells were centrifuged at 600 g at 4°C for 3 min. The supernatant was discarded, and then 1 × JC-1 staining buffer was added to resuspend the cells, which were analyzed by flow cytometry (Becton-Dickinson, Franklin Lakes, NJ, United States). Mitochondrial depolarization was evaluated by detecting the excitation wavelengths of JC-1 (488 nm), and J-aggregate forms were assessed at 529 and 590 nm.

### Reactive Oxygen Species (ROS) Assays

Intracellular reactive oxygen species (ROS) production was detected using the redox-sensitive dye DCFH-DA (Beyotime Biotech, Nanjing, China). U251 cells (1 × 10^5^ cells/well) were plated in 24-well microplates, incubated overnight, and then treated with 2 μM sorafenib and 100 μM TMZ in the absence or presence of NAC (ROS scavenger). The control was cultured with medium containing an appropriate amount of DMSO. All the experimental cells were washed 3 times with cold phosphate-buffered saline (PBS) and then incubated with dichlorodihydrofluorescein diacetate (DCFH-DA) (Sigma) at 37°C for 15 min. After the cells were washed 3 times with PBS, the ROS levels were measured by fluorescence microscopy (Olympus IX71, Tokyo, Japan). Fluorescence was detected at an excitation wavelength of 488 nm and an emission wavelength of 525 nm.

### TUNEL Assay

Cell apoptosis was evaluated by a TUNEL assay. Cells were labeled with an *In Situ* Cell Death Detection Kit, Fluorescein (Roche Diagnostics, Mannheim, Germany) and then treated with 2 μM sorafenib for 12 and 24 h. The control was cultured with medium containing an appropriate amount of DMSO. Approximately 1 × 10^6^ cells were collected, washed once with PBS, resuspended, and added to the polylysine slide. The slides were fixed with 4% paraformaldehyde for 25 min and then washed with PBS. The slides were incubated with 0.2% Triton X-100 for 5 min and washed with PBS. After the slides were dried, 50 μl of TUNEL reaction mixture was added to the slides. Then, the slides were incubated at 37°C for 1 h in a dark wet box and washed with PBS 3 times. The cells were then analyzed with a fluorescence microscope (Olympus IX71, Tokyo, Japan).

### Immunohistochemical Staining

Immunohistochemical staining was performed on glioma xenograft models. Glioma xenograft tumor tissues were fixed, embedded in paraffin and cut into 4-μm sections. Using a graded alcohol series, the sections were deparaffinized with xylene and dehydrated. Antigen retrieval was performed by microwaving in pH 6.0 citrate buffer. Then, the sections were immunostained with primary antibodies against p-JAK2 (1:400, Abcam, United States) and p-STAT3 (1:100, Cell Signaling Technology, United States) in a humidified container and visualized using diaminobenzidine (DAB) staining. The stained cells that primarily showed brown signals in the cytoplasm indicated positive reactions. Finally, the images were visualized at 40× magnification with a Leica microscope.

### Glioma Tumor Xenografts in Mice

Athymic BALB/c nude mice (4–6 weeks old and weighing 19–21 g) were purchased from Beijing Vital River Laboratory Animal Technology Co., Ltd., and allowed to acclimate to their surroundings for 3 days, with food and water provided *ad libitum*. The glioma tumor xenograft model was established by the subcutaneous injection of 1 × 10^6^ logarithmically grown U251 cells in 100 μl of PBS into the right flank of each mouse. Therapeutic experiments with the glioma tumor xenograft model were started when the tumor volume reached approximately 160–210 mm^3^. The mice were randomly divided into four groups (*n* = 5 per group) and administered TMZ (25 mg/kg mouse weight, intraperitoneal injection) alone, sorafenib (20 mg/kg mouse weight, by gavage) alone, TMZ and sorafenib in combination, or vehicle as a control every other day. The tumor volume was calculated according to the following formula: length × width × height × 0.5. The diameter was measured by a caliper (Ma et al., 2019). After the mice were euthanized, the tumor tissues were excised and measured.

### Statistical Analysis

Statistical significance was determined based on *P*-values less than 0.05. The error bars for all data represent the mean ± SD from at least three independent experiments. Paired Student’s *t*-test was used to compare treatments, and multiple group comparisons with single controls were performed using one-way ANOVA. SPSS version 16.0 (SPSS/IBM, Chicago, Illinois, United States) was used for the analyses.

## Results

### Sorafenib Inhibited the Viability and Induced the Apoptosis of Glioma Cell Lines

**FIGURE 1:**
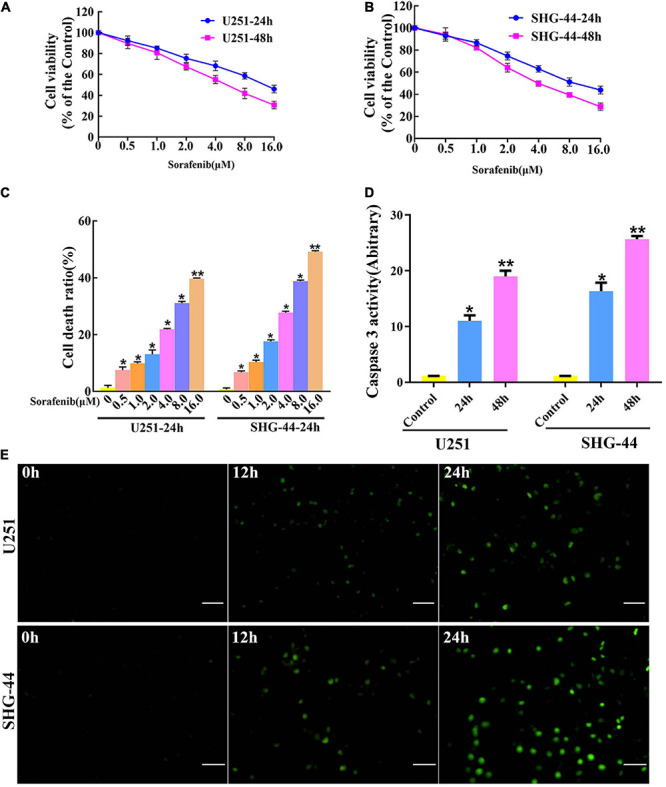
Sorafenib inhibits the growth of glioma cell lines. **(A,B)** Glioma cell lines, including U251 and SHG-44, were treated with varying concentrations of sorafenib for 24 or 48 h. Cell viability was determined by MTT assay, and the results showed that sorafenib significantly decreased the viability of glioma cells in a time- and dose-dependent manner. Data are presented as the mean ± SD (*n* = 3). **(C)** Cells were treated with varying sorafenib concentrations for 24 h, and cells were detected by flow cytometry analysis with Annexin V and PI double staining and quantitated. Flow cytometry analysis confirmed that dose-dependent death was induced by sorafenib in U251 and SHG-44 cells (*P < 0.05 and **P < 0.05 versus control group). **(D,E)** U251 and SHG-44 cells treated with 2 μM sorafenib. **(D)** Activity of caspase 3 was measured by a colorimetric assay kit. The results showed that sorafenib increased caspase 3 enzyme activity in U251 and SHG-44 cells in a time-dependent manner (*P < 0.05 and **P < 0.05 versus control group). **(E)** TUNEL assay with confocal microscopy showed that sorafenib induced apoptosis of U251 and SHG-44 cells in a time-dependent manner (20×) (Scale bar = 200 μm).

To evaluate the toxic effect of sorafenib on malignant glioma cells, glioma cells were treated with different doses of sorafenib for 24 or 48 h. MTT assays showed that sorafenib significantly decreased the viability of C6, U251, LN18, and SHG-44 glioma cells in a time- and dose-dependent manner (Figures 1A,B and Supplementary Figure 1). In particular, after treatment with sorafenib at the indicated concentrations for 24 h, the viability of C6, U251, LN18, and SHG-44 glioma cells was drastically decreased. Thus, the 24 h IC50 values of sorafenib were 9.1 μmol/L for C6 cells, 13.8 μmol/L for U251 cells, 10.7 μmol/L for IN18 cells, and 9.8 μmol/L for SHG-44 cells. To assess the death of glioma cells caused by sorafenib, glioma cells were stained with Annexin V-FITC and PI dual staining and analyzed using flow cytometry. As shown in Figure 1C, different concentrations of sorafenib significantly induced U251 and SHG-44 glioma cell death at 24 h. Next, we treated the cells with 2 μM sorafenib for various time points and analyzed the cells using caspase 3 enzyme activity assays (Figure 1D) and TUNEL assays (Figure 1E). The results showed that sorafenib resulted in increased caspase 3 enzyme activity and DNA fragmentation in U251 and SHG-44 glioma cells in a time-dependent manner. These results suggest that sorafenib inhibited the growth and induced the apoptosis of glioma cells in a time- and dose-dependent manner.

### Sorafenib Synergistically Enhanced the Effects of TMZ on Cell Proliferation and Cell Apoptosis in Glioma Cell Lines

**FIGURE 2:**
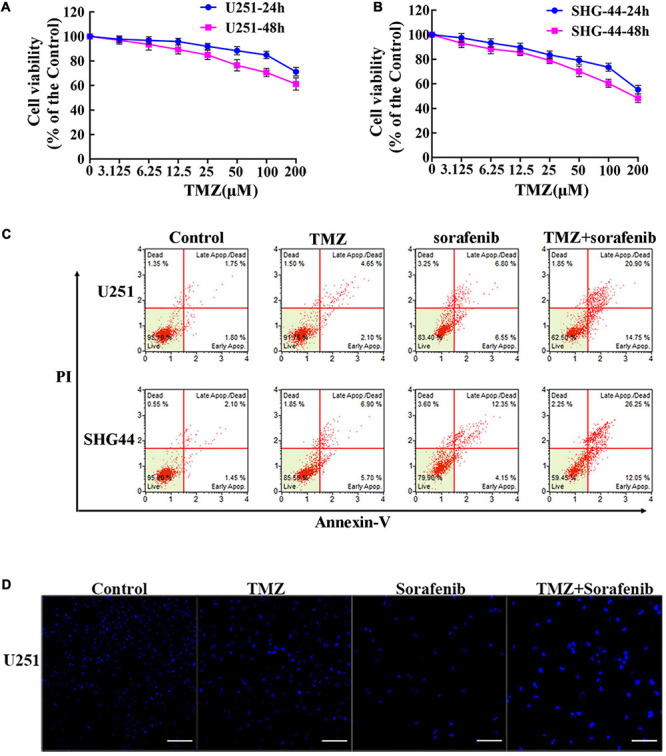
Sorafenib enhanced TMZ cytotoxicity in glioma cell lines. **(A,B)** Glioma cell lines, including U251 and SHG-44, were treated with varying concentrations of temozolomide (abbreviated as TMZ) for 24 or 48 h. MTT assay showed that TMZ significantly decreased the viabilities of glioma cells in a time- and dose-dependent manner. **(C,D)** U251 and SHG-44 cells were treated with 2 μM sorafenib and 100 μM TMZ for 24 h. **(C)** Flow cytometry with Annexin V and PI double staining showed that the combination of sorafenib and TMZ strikingly induced the apoptosis of glioma cells. **(D)** Cells stained with Hoechst 33342 demonstrated that the combination of sorafenib and TMZ induced apoptotic changes to chromatin in U251 cells after 24 h (20×) (Scale bar = 10 μm).

To evaluate the effect of TMZ on malignant glioma cells, we treated the cells with different concentrations of TMZ. As shown in Figures 2A,B and Supplementary Figures 2A,B, TMZ significantly decreased the viability of the C6, U251, LN18, and SHG-44 glioma cells in a time- and dose-dependent manner. We then evaluated whether this effect was synergistic, additive, or antagonistic when the U251 and SHG-44 glioma cells were treated with a combination of 2 μM sorafenib and 100 μM TMZ. According to MTT assay (Supplementary Figure 2C), the combination of sorafenib and TMZ had a synergistic effect on the U251 and SHG-44 glioma cells for 24 h. To further assess the role of sorafenib and TMZ, we quantified the percentage of apoptosis using Annexin V/PI staining and flow cytometry. After incubation for 24 h, the combination of sorafenib and TMZ strikingly induced the apoptosis of glioma cells compared with the single agents alone (*P* < 0.05) (Figure 2C). To further determine the effect of the combination of sorafenib and TMZ on nuclear morphology, we used Hoechst 33342 staining in U251 cells. The combination of sorafenib and TMZ induced more apoptotic changes to chromatin in glioma cells than either sorafenib or TMZ alone (Figure 2D). These results suggest that the combination of sorafenib and TMZ enhanced the antitumor effect of TMZ in glioma cell lines.

### Combination of Sorafenib and TMZ Induced Mitochondrial Depolarization and Nuclear AIF Aggregation

**FIGURE 3:**
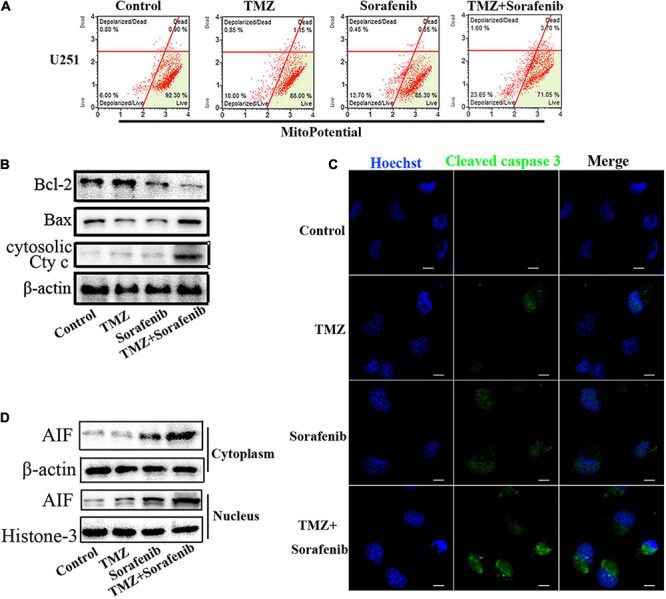
Combination of sorafenib and TMZ triggered mitochondrial damage and nuclear AIF aggregation. **(A)** After treating U251 cells with a combination of sorafenib and TMZ for 24 h, the mitochondrial membrane potential was stained with JC-1 and assessed *via* flow cytometry analysis. The results showed that the combination of sorafenib and TMZ decreased the mitochondrial membrane potential of U251 cells. **(B)** Western blot assay of Bcl-2, Bax, and Cyt c protein expression in U251 cells treated with the combination of sorafenib and TMZ for 24 h. Western blot assay demonstrated that the combination of sorafenib and TMZ upregulated the expression of Bax and Cyt c and downregulated the level of Bcl-2. **(C)** Representative images of U251 cells with confocal microscopy showed that the combination of sorafenib and TMZ increased the expression of cleaved caspase 3 (120×) (bar, 10 μm). **(D)** Western blot assay of the expression of cytoplasmic and nuclear AIF protein in U251 cells for 24 h. The results showed that the combination of sorafenib and TMZ increased the nuclear aggregation of AIF.

Mitochondrial function plays an important role in cell apoptosis. Rapamycin has a synergistic effect with Temozolomide in inducing apoptosis of human glioblastoma cells throughmitochondrial dysfunction (Zimmerman et al., 2020). And sorafenib has an effect in mitochondrial respiratory machinery (Bull et al., 2012). To determine the mechanism underlying the ability of the combination of sorafenib and TMZ to induce apoptosis, we detected changes in mitochondrial membrane potential via flow cytometry analysis. As shown in Figure 3A, the combination of sorafenib and TMZ obviously decreased the mitochondrial membrane potential of U251 cells compared to sorafenib or TMZ alone. The decreased mitochondrial membrane potential led to an abnormal ratio of Bax and Bcl-2 and the release of cytosolic cytochrome c from the mitochondria. Next, we examined the protein levels of Bax, Bcl-2 and cytosolic cytochrome c by Western blotting. As shown in Figure 3B and Supplementary Figure 3A, the expression of Bax and cytosolic cytochrome c was increased in the U251 cells treated with the combination of sorafenib and TMZ compared with the cells treated with either agent alone. Conversely, the level of Bcl-2 was obviously downregulated after treatment with the combination of sorafenib and TMZ. Fluorescence microscopy of cleaved caspase 3 indicated that this protein was significantly enhanced in the U251 cells treated with the combination of sorafenib and TMZ compared with the cells treated with either agent alone (Figure 3C). Furthermore, the decreased mitochondrial membrane potential could lead to AIF release and nuclear aggregation (Zhao et al., 2016). We found that the nuclear aggregation of AIF was consistent with the upregulation of Bax and the downregulation of Bcl-2 in U251 glioma cells (Figure 3D and Supplementary Figure 3B). These results suggest that the combination of sorafenib and TMZ triggered glioma cell apoptosis through the mitochondrial-associated pathway and was associated with the nuclear aggregation of AIF.

### AIF Contributed to Apoptosis Induced by the Combination of Sorafenib and TMZ in Glioma Cells

**FIGURE 4:**
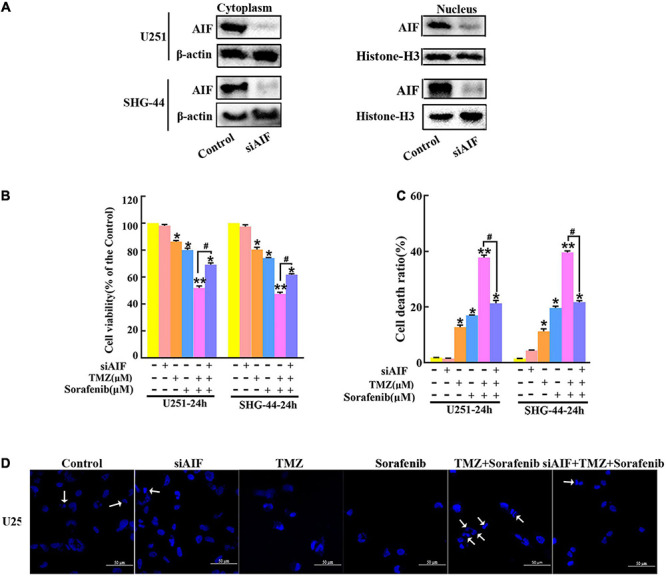
Combination of sorafenib and TMZ-induced apoptosis is associated with AIF nuclear aggregation. **(A–D)** AIF was knocked down in U251 and SHG-44 cells using small interfering RNA (siRNA). **(A)** After AIF was knocked down, Western blot assays showed that AIF siRNA downregulated the cytoplasmic and nuclear levels of AIF in U251 and SHG-44 cells. **(B)** After cells were treated with the combination of sorafenib and TMZ and siAIF, the MTT assay proved that siAIF decelerated the reduction in U251 and SHG-44 cells viability induced by the combination of sorafenib and TMZ (*P < 0.05 and **P < 0.01 versus the control group, ^#^*P* < 0.05 versus the combination of sorafenib and TMZ). **(C)** After the same treatment as **(B)**, flow cytometry analysis with Annexin V and PI double staining confirmed that siAIF reversed the combination of sorafenib and TMZ-induced U251 and SHG-44 cell death (*P < 0.05 and **P < 0.01 versus the control group, ^#^*P* < 0.05 versus the combination of sorafenib and TMZ). **(D)** Representative images of U251 cells stained with Hoechst 33342 using a fluorescence microscope showed that siAIF mitigated the combination of sorafenib and TMZ-induced apoptotic changes to chromatin (20×) (Scale bar = 50 μm).

AIF normally exists between the interior and external mitochondrial membranes, and acts as an executioner under the stimulation of endogenous or exogenous apoptotic signals (Zhang et al., 2021). To evaluate the role of AIF in the apoptosis induced by the combination of sorafenib and TMZ in glioma cells, we knocked down AIF using small interfering RNA. Western blot assays showed that cytoplasmic and nuclear AIF expression was decreased in U251 and SHG-44 glioma cells treated with AIF siRNA (Figure 4A and Supplementary Figures 4A,B). In addition, the results of the MTT assay showed that the combination of sorafenib and TMZ reduced U251 and SHG-44 glioma cell viability, which was partially reversed by knocking down AIF (Figure 4B). Furthermore, the optical electron microscopy results were also consistent with the MTT results of U251 cells (Supplementary Figure 4C). Consistent with the MTT results, a similar phenomenon was observed by flow cytometry in the U251 and SHG-44 glioma cells (Figure 4C). To further determine the effect of the combination of sorafenib, TMZ and siAIF on nuclear morphology, we performed Hoechst 33342 staining of U251 glioma cells. Apoptotic changes to chromatin in the glioma cells treated with the combination of sorafenib and TMZ were reduced when AIF was knocked down by siRNA (Figure 4D and Supplementary Figure 4D). These results suggest that AIF plays an important role in apoptosis induced by the combination of sorafenib and TMZ.

### Inhibition of STAT3 Promoted Mitochondrial Depolarization and AIF Nuclear Aggregation

**FIGURE 5:**
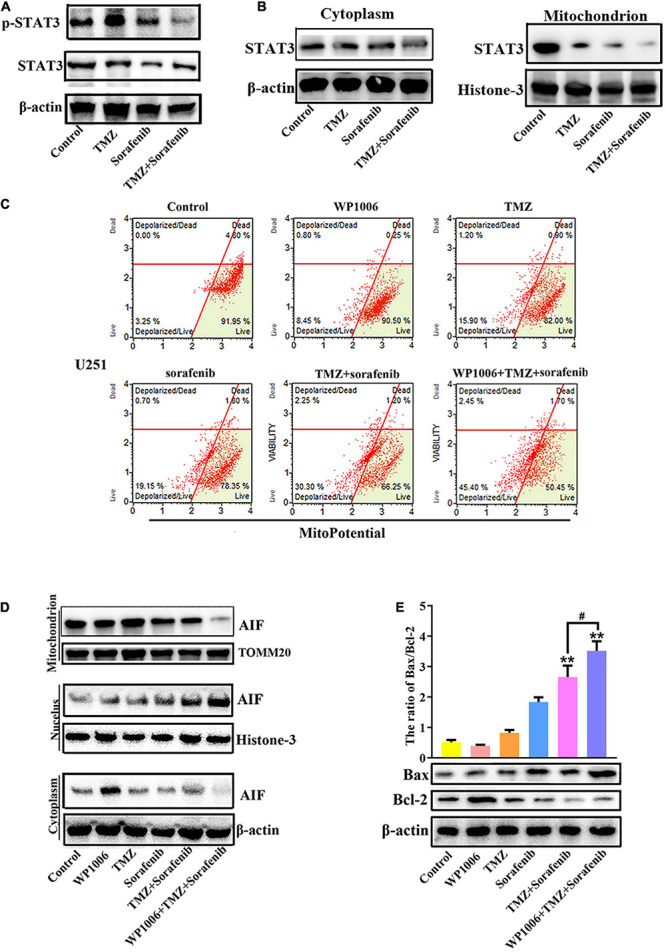
Combination of sorafenib and TMZ-induced mitochondrial depolarization and AIF nuclear aggregation is associated with STAT3 inhibition. **(A)** Western blot analysis revealed that the level of p-STAT3 protein was downregulated in U251 cells treated with the combination of sorafenib and TMZ for 24 h. **(B)** Western blot assays revealed that the combination of sorafenib and TMZ reduced the expression of mitochondrial STAT3 protein in U251 cells. **(C)** After U251 cells were treated with WP1006 and the combination of sorafenib and TMZ for 24 h, flow cytometry analysis with JC-1 staining showed that WP-1006 enhanced the mitochondrial depolarization induced by the combination of sorafenib and TMZ in U251 glioma cells. **(D)** Western blot assays revealed that WP-1006 accelerated the sorafenib and TMZ treatment-induced translocation of AIF from mitochondria to nuclei in U251 cells. **(E)** After the same treatment as **(D)**, Western blot assay and quantitation showed that WP-1006 increased the ratio of the protein Bax/Bcl-2 induced by the combination of sorafenib and TMZ in U251 cells (**P < 0.01 compared to the control group, ^#^*P* < 0.05 versus the combination of sorafenib and TMZ).

Constitutive STAT3 activation plays an important role in driving tumorigenesis and tumor cell death resistance, and the inhibition of STAT3 activation in a tumor is involved in mitochondrial depolarization and mitochondrial-associated apoptotic pathways (Yuan et al., 2015). To better understand the mechanism by which the combination of sorafenib and TMZ induced mitochondrial depolarization and AIF nuclear aggregation, we assessed the expression and activation of STAT3 and found that the combination of sorafenib and TMZ led to greater inhibition of STAT3 phosphorylation compared with either agent alone (Figure 5A and Supplementary Figure 5A). Meanwhile, the combination of sorafenib and TMZ significantly inhibited STAT3 translocation to mitochondria (Figure 5B). Next, we inhibited STAT3 by adding WP-1006, a STAT3 inhibitor, and analyzed mitochondrial depolarization and AIF translocation from mitochondria to nuclei. As shown in Figures 5C,D and Supplementary Figures 5B–D, we found that inhibition of STAT3 with WP-1006 enhanced the mitochondrial depolarization and AIF translocation from mitochondria to nuclei induced by the combination of sorafenib and TMZ in U251 glioma cells. Furthermore, we found that inhibition of STAT3 enhanced the upregulation of Bax and downregulation of Bcl-2 in glioma cells treated with the combination of sorafenib and TMZ (Figure 5E). These results suggest that the combination of sorafenib and TMZ inhibited STAT3 activation and that STAT3 inhibition promoted mitochondrial depolarization and AIF translocation from mitochondria to nuclei to induce cell apoptosis.

### Inhibition of STAT3 Promoted the Autophagy-Associated Apoptosis Induced by the Combination of Sorafenib and TMZ in Glioma Cells

**FIGURE 6:**
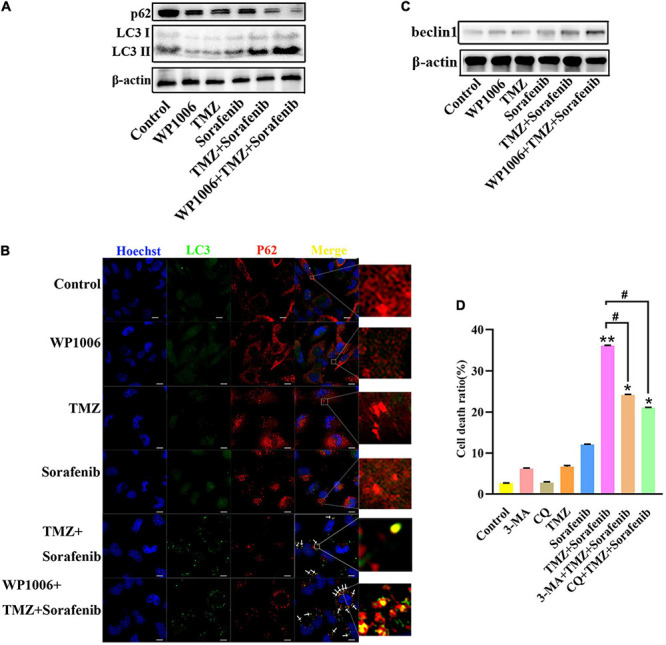
Combination of sorafenib and TMZ-induced autophagy is associated with the inhibition of STAT3. **(A)** Western blot analysis showed that the combination of sorafenib and TMZ upregulated the level of LC3II and downregulated the expression of p62. Meanwhile, WP-1006 enhanced sorafenib and TMZ treatment-induced autophagy of U251 cells. **(B)** After the same treatment as **(A)**, representative images under a fluorescence microscope revealed that the combination of sorafenib and TMZ induced the colocalization of p62 and LC3 at 12 h and WP-1006 enhanced the colocalization induced by the combination of sorafenib and TMZ in U251 cells (120×) (bar, 10 μm). **(C)** After the same treatment as **(A)**, Western blot assay showed that the combination of sorafenib and TMZ increased the expression of autophagy-related protein Beclin 1 and WP-1006 promoted this upregulation of Beclin 1 in U251 cells. **(D)** After U251 cells were treated with 3-MA or CQ and the combination of sorafenib and TMZ, flow cytometry analysis with Annexin V and PI double staining showed that 3-MA or CQ prevented cell apoptosis induced by the combination of sorafenib and TMZ (*P < 0.05 and **P < 0.01 compared to the control group, ^#^*P* < 0.05 versus the combination of sorafenib and TMZ).

Because sorafenib can induce autophagy in tumors (Nawara et al., 2020) and STAT3 is associated with autophagy, we investigated the changes in autophagy induced by the combination of sorafenib and TMZ. As shown in Figure 6A and Supplementary Figure 6A, LC3-II expression showed a greater increase in U251 cells treated with the combination of sorafenib and TMZ than in cells treated with either agent alone. Conversely, the combination obviously downregulated the expression of the protein p62. Moreover, we found that an inhibitor of STAT3, WP-1006, enhanced the autophagy induced by the combination of sorafenib and TMZ in U251 cells. WP-1006 enhanced the downregulation of p62 and upregulation of LC3II in U251 cells treated with the combination of sorafenib and TMZ (Figure 6A and Supplementary Figure 6A). Meanwhile, we found that WP-1006 could enhance the colocalization of p62 and LC3 induced by the combination of sorafenib and TMZ at 12 h in U251 glioma cells (Figure 6B). WP-1006 also promoted the upregulation of the autophagy-related protein Beclin 1 induced by the combination of sorafenib and TMZ in U251 glioma cell lines (Figure 6C and Supplementary Figure 6B). To better understand the role of autophagy in the decreased cell apoptosis mediated by the combination of sorafenib and TMZ, we treated glioma cells with autophagy inhibitors. As shown in Figure 6D, when we treated U251 and SHG-44 glioma cells with 3-MA (5 mmol/l), a specific inhibitor of the early stages of the autophagic process, or CQ, an autophagy inhibitor that increases lysosomal pH levels and blocks autophagosome–lysosome fusion. 3-MA or CQ prevented the apoptosis of U251 glioma cells induced by the combination of sorafenib and TMZ. These results suggest that the combination of sorafenib and TMZ enhances autophagic flux and that inhibition of STAT3 enhances the level of autophagy in glioma cells. Autophagy promoted the apoptosis of glioma cells induced by the combination of sorafenib and TMZ.

### ROS Regulated the JAK2/STAT3 Pathway in Glioma Cells Treated With the Combination of Sorafenib and TMZ

**FIGURE 7:**
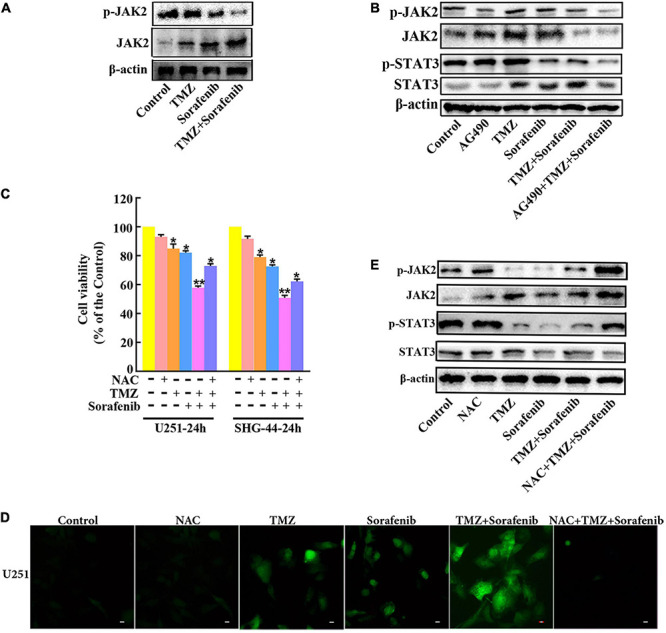
Inhibition of the JAK2/STAT3 pathway in glioma cells treated with the combination of sorafenib and TMZ is associated with ROS. **(A)** Western blot analysis showed that the combination of sorafenib and TMZ decreased the level of phosphorylated JAK2 in U251 cells. **(B)** After U251 cells were treated with AG490 and the combination of sorafenib and TMZ for 24 h, Western blot showed that AG490 decreased the levels of phosphorylated JAK2 and STAT3 in U251 cells induced by the combination of sorafenib and TMZ. **(C)** After U251 cells were pretreated with NAC, MTT assay showed that NAC reversed the reduced viability caused by the combination of sorafenib and TMZ in U251 cells (*P < 0.05 and **P < 0.01 versus the control group). **(D)** After the same treatment as **(C)**, representative images of U251 cells stained with DCFH-DA under a fluorescence microscope showed that the combination of sorafenib and TMZ increased the level of ROS and NAC suppressed this abnormal elevation of intracellular ROS induced by the combination of sorafenib and TMZ. **(E)** After U251 cells were treated with NAC and the combination of sorafenib and TMZ for 24 h, Western blot analysis showed that NAC reversed the phosphorylation of JAK2 and STAT3 inhibited by the combination of sorafenib and TMZ.

To determine the mechanism by which STAT3 was inhibited by the combination of sorafenib and TMZ, we assessed JAK2, an upstream molecule that regulates STAT3 in glioma cells. As shown in Figure 7A and Supplementary Figure 7A, the combination of sorafenib and TMZ significantly decreased the expression of phosphorylated JAK2 in U251 glioma cells. Meanwhile, we inhibited the activation of JAK2 with the JAK2 inhibitor AG490 and found that AG490 significantly decreased the levels of phosphorylated JAK2 and STAT3 in U251 cells treated with the combination of sorafenib and TMZ (Figure 7B and Supplementary Figure 7B). Previous reports have revealed that ROS are involved in the JAK2/STAT3 pathway (Cao et al., 2020); thus, we examined the effect of ROS on the JAK2/STAT3 pathway. As shown in Figure 7D, intracellular ROS were overproduced in U251 cells treated with the combination of sorafenib and TMZ compared with cells treated with either single agent alone. In contrast, NAC, a ROS scavenger, not only significantly suppressed the abnormal elevation in intracellular ROS induced by the combination of sorafenib and TMZ (Figure 7D) in U251 cells but also reversed the reduced viability caused by the combination of sorafenib and TMZ in U251 cells (Figure 7C). Moreover, NAC significantly reversed the phosphorylation of JAK2 and STAT3 inhibited by the combination of sorafenib and TMZ in U251 cells (Figure 7E and Supplementary Figure 7C). These results suggest that the combination of sorafenib and TMZ could induce ROS and that ROS played an important role in cell apoptosis and inhibition of JAK2/STAT3 induced by the combination of sorafenib and TMZ in glioma cells.

### Combination of Sorafenib and TMZ Inhibited the Growth of Gliomas *in vivo* in Xenograft Models

**FIGURE 8:**
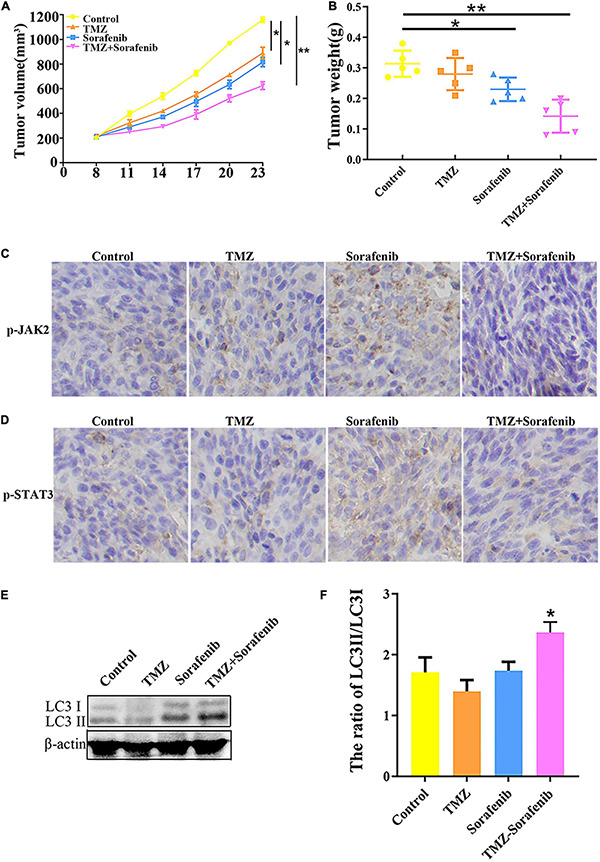
Combination of sorafenib and TMZ inhibited the growth of gliomas *in vivo*. Tumors originating from U251 cells were treated with a combination of sorafenib and TMZ. **(A)** Combination of sorafenib and TMZ decreased the average tumor volume of xenograft gliomas in nude mice (*P < 0.05 and **P < 0.01 versus the control group). **(B)** Combination of sorafenib and TMZ decreased the average tumor weights of xenograft gliomas in nude mice (*P < 0.05 and **P < 0.01 versus the control group). **(C,D)** Immunohistochemistry showed that the combination of sorafenib and TMZ inhibited the expression of p-JAK2 and p-STAT3 in U251 xenograft tumor tissues (40×). **(E,F)** Western blot analysis and quantitation showed that the combination of sorafenib and TMZ increased the level of LC3II in U251 xenograft tumors (*P < 0.05 versus control group).

To examine the effect of the combination of sorafenib and TMZ *in vivo*, a xenograft glioma model was established using the U251 cell line. As shown in Figures 8A,B, both the average volume and the average weight of the gliomas were obviously decreased in the group treated with the combination of sorafenib and TMZ compared with the groups treated with sorafenib or TMZ alone. In addition, we found that the levels of phosphorylated JAK2/STAT3 observed by immunohistochemistry were significantly decreased in the group treated with the combination of sorafenib and TMZ compared with the groups treated with sorafenib or TMZ alone (Figures 8C,D). Simultaneously, we found that the combination of sorafenib and TMZ significantly increased the expression of LC3II compared with sorafenib or TMZ alone (Figures 8E,F). These results suggest that sorafenib could enhance the antiglioma effect of TMZ and promote the apoptosis of glioma cells through the JAK2/STAT3 and autophagy pathways.

## Discussion

Malignant glioma is the most common type of primary malignant brain tumor, and it is associated with high rates of morbidity and recurrence (Zhang and Liu, 2020). TMZ is a traditional chemotherapy drug used in the treatment of glioma; however, TMZ resistance is the main factor that leads to glioma treatment failure (Liu et al., 2020). Therefore, enhancing the sensitivity of glioma cells to TMZ has become the focus of clinical research. Reports show that sorafenib has the ability to effectively cross the blood-brain barrier and is well tolerated after systemic administration (Siegelin et al., 2010; Nabors et al., 2011). In our study, we found that glioma cells are not resistant to sorafenib but are resistant to TMZ. Moreover, sorafenib could enhance the cytotoxicity of TMZ in glioma cells *in vitro* and *in vivo*. The combination of sorafenib and TMZ inhibited the viability of glioma cells *in vitro* and the growth of xenograft gliomas *in vivo*. Consistently, the combination of sorafenib and temozolomide showed activity in patients with relapsed GBM, although the mechanism has not been clarified (Zustovich et al., 2013). Meanwhile, sorafenib and temozolomide synergistically induced programmed cell death in MOGGCCM and T98G cells, although the effectiveness of both drugs was cell-type specific (Jakubowicz-Gil et al., 2017). However, a previous study reported that sorafenib failed to enhance the death of GBM cells caused by irradiation, TMZ or combined treatment and caused resistance in some cell lines (Riedel et al., 2016), and these findings are partially inconsistent with our conclusions. We speculate that different cell types and drug treatment methods may lead to different results. Therefore, it is very important to clarify the mechanism by which sorafenib enhances the sensitivity of glioma cells to TMZ. In our study, we found that the combination of sorafenib and TMZ simultaneously triggered apoptosis through the induction of oxidative stress-mediated autophagy and JAK2/STAT3-AIF axis.

Sorafenib is an inhibitor of serine/threonine kinases in the Ras-Raf-MEK-ERK pathway and affects angiogenesis- and tumorigenesis-related pathways through the inhibition of several kinases, including VEGFR1, VEGFR2, VEGFR3, PDGFRβ, c-Kit, and RET2 (Hage et al., 2019; Cabral et al., 2020). Sorafenib can target mitochondrial respiratory machinery processes and damage mitochondrial metabolic functions (Bull et al., 2012; Zhang et al., 2017). The Bcl-2 network is involved in cell apoptosis via mitochondrial outer membrane permeabilization (MOMP) through the proapoptotic proteins BAX and BAK (Kuwana et al., 2020; Solà-Riera et al., 2020). MOMP leads to the release of pro-apoptotic cytochrome c and smac/DIABLO, which induces the activation of executioner caspases and cell death (Vicinanza and Rubinsztein, 2020). In the present study, the combination of sorafenib and TMZ obviously decreased the mitochondrial membrane potential of glioma cells (Figure 3A) and increased the Bax/Bcl-2 ratio and cytosolic cytochrome c release (Figure 3B). Interestingly, the combined therapy could induce AIF release and nuclear aggregation (Figure 3D). Moreover, inhibition of AIF by knockdown could significantly decrease the apoptosis induced by the combined therapy in glioma cells (Figures 4B–D). A recent study indicated that sorafenib could sensitize resistant HCC cells to radiation through inhibition of the STAT3-associated pathway *in vitro* and *in vivo* (Huang et al., 2013). Mcl-1 and Bcl-xL are involved in the prosurvival pathway and represent target genes of STAT3 (Radhakrishnan et al., 2017; Yan et al., 2018). Correspondingly, we found that the combined therapy inhibited the activation and mitochondrial localization of STAT3 (Figures 5A,B). Meanwhile, inhibition of STAT3 enhanced the combined therapy-induced mitochondrial depolarization and AIF translocation from mitochondria to nuclei (Figures 5D,E). In summary, the combination of sorafenib and TMZ induces cell apoptosis through STAT3-dependent nuclear translocation of AIF.

In this study, we found that the combined therapy could induce autophagy in glioma cells. Autophagy has been extensively studied as a modulator of pathogenesis in many diseases (Crawley et al., 2019; Silva et al., 2020). We confirmed that the combined therapy triggered autophagy, increased Beclin1 and LC3II expression and decreased p62 expression (Figure 6). As an inhibitor of STAT3, WP006 alone did not significantly change LC3II but significantly upregulated the expression of LC3II and Beclin 1 (Figures 6A,C) and induced a greater number of yellow autophagic vesicles (Figure 6B) in the combination of sorafenib and TMZ. This result may indicate that combined therapy induced autophagy through the inhibition of STAT3 and activation of Beclin 1 (Maycotte et al., 2014). We further confirmed that the autophagy inhibitors 3-MA and CQ moderately reversed the inhibitory effect of the combination of sorafenib and TMZ on the cell apoptosis rate (Figure 6D), indicating that the combined therapy induces cell apoptosis through the induction of autophagy. Autophagy and apoptosis can trigger cell death through synergistic action and complementary cooperation (París-Coderch et al., 2020). The proapoptotic effect of autophagy caused by the combined therapy also indicated that autophagy and apoptosis caused by combined therapy had synergistic effects.

In glioma cells, anticancer drugs often upregulate the level of ROS, thereby triggering cell signaling and even cell death (Yang et al., 2020). In our study, the combined therapy aggravated the aggregation of ROS (Figure 7D). The accumulation of ROS could inactivate STAT3 activity in renal cell carcinoma (RCC) (He et al., 2020). STAT3 activation can be induced by the upstream tyrosine kinases Src and JAK (Zhou et al., 2020), whose inhibition blocks STAT3 signaling activation in melanoma (Zhu et al., 2020). Our study showed that the combination of sorafenib and TMZ also decreased the phosphorylation of JAK2 (Figure 7A) and that blocking the activation of JAK2 by AG490 obviously decreased the phosphorylation of STAT3 (Figure 7B). In addition, NAC obviously attenuated the phosphorylation of JAK2 and STAT3 (Figure 7E), and in the cells treated with the combination of sorafenib and TMZ, ROS generation was significantly decreased after pretreatment with NAC (Figure 7D). An inhibitory effect may exist between ROS and the JAK2/STAT3 signaling pathway. Taken together, the above results suggested that the combination of sorafenib and TMZ induces cell death, which may be mediated through the generation of oxidative stress and inhibition of the JAK2/STAT3 signaling pathway.

**FIGURE 9:**
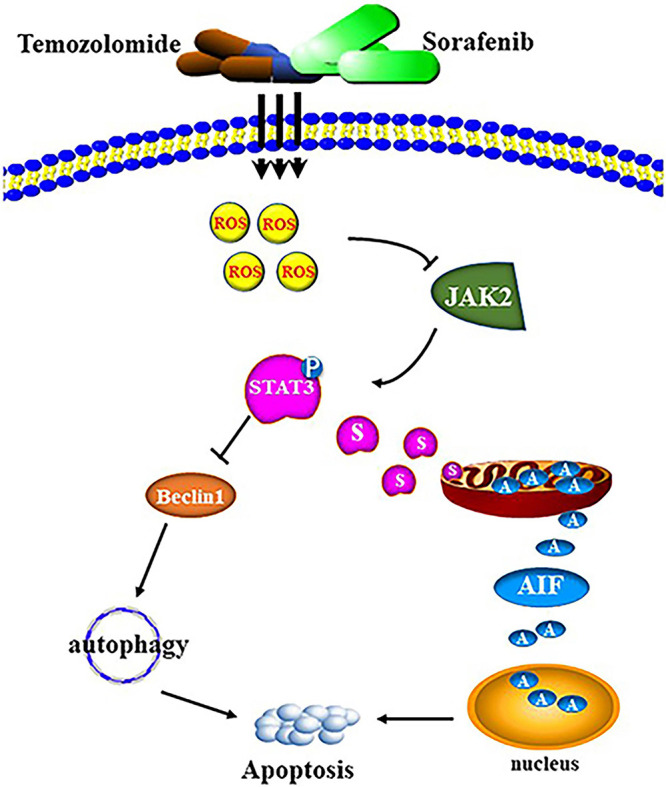
Schematic diagram of cell apoptosis induced by the combination of sorafenib and TMZ.

The combination of sorafenib and TMZ also inhibited the growth of tumors *in vivo*, as observed with a xenograft glioma model in nude mice (Figures 8A,B). The results showed that xenograft gliomas are resistant to TMZ but not to combined therapy when compared with the control. Furthermore, immunohistochemical analysis and Western blotting showed that the combined therapy significantly inhibited the activation of JAK2 and STAT3 but promoted the induction of autophagy (Figures 8C–F). However, we did not inject glioma cells intracranially into nude mice and the mechanisms underlying the effects of sorafenib and TMZ in orthotopic GBM models need to be clarified. The *in vitro* data suggested that the combination therapy inhibited the tumor formation ability of glioma. In conclusion, our study confirmed that sorafenib enhanced the temozolomide sensitivity of human glioma cells through oxidative stress and JAK2/STAT3 signaling pathway inhibition. Further research indicated that STAT3 activation inhibition induced AIF translocation from mitochondria to nuclei and promoted autophagy through Beclin 1 (Figure 9).

## Conclusion

Collectively, our study confirmed that sorafenib enhanced the temozolomide sensitivity of human glioma cells through oxidative stress-mediated autophagy and JAK2/STAT3-AIF axis. It provides insights into the activities of the combined therapy and indicate a potential therapeutic strategy for the treatment of glioma.

## Data Availability Statement

The original contributions presented in the study are included in the article/Supplementary Material, further inquiries can be directed to the corresponding author/s.

## Ethics Statement

The animal study was reviewed and approved by the First Affiliated Hospital of Zhengzhou University.

## Author Contributions

JW designed the experiments and wrote the manuscript. ZW and WW performed the immunoblotting and immunofluorescence assays. JW and XLi performed the RNA interference experiments. YY and XLi established the animal models. LM and XLiu conceived the concept. XLiu analyzed the data. All authors read and approved the final manuscript.

## Conflict of Interest

The authors declare that the research was conducted in the absence of any commercial or financial relationships that could be construed as a potential conflict of interest.

## Abbreviations

ROS: reactive oxygen species
TMZ: temozolomide
MTT: (3-(4,5-dimethylthiazol-2-yl)-2,5-diphenyltetrazolium bromide
DCFH-DA: dichlorodihydrofluorescein diacetate
PBS: phosphate-buffered saline
MMP: ΔΨ m, mitochondrial membrane potential
STAT3: Signal transductor and transcriptional activator 3; CytC (Citrochrome C)
NAC: N-acetyl -l-cysteine
MOMP: mitochondrial outer membrane permeabilization
DAB: diaminobenzidine.
