# Supplementary material for: Oxidative Stress Activated by Sorafenib Alters the Temozolomide Sensitivity of Human Glioma Cells Through Autophagy and JAK2/STAT3-AIF Axis
Source: Front Cell Dev Biol. 2021 Jun 14;9:660005. doi: 10.3389/fcell.2021.660005 (PMC8282178; doi:10.3389/fcell.2021.660005)
Supplement: Supplementary file 1 [file Table_1.docx]

**Supplementary Figure legends**

**Oxidative stress activated by** **sorafenib alters the temozolomide sensitivity of human glioma cells through autophagy and JAK2/STAT3-AIF axis**

Jianwei Wei ^1^, zhengfeng wang^1^, Weiwei Wang ^2^, Xiaoge Liu ^3^, Junhu Wan ^4^, Yongjie Yuan ^5^, Xueyuan Li ^1^, Liwei Ma ^4^*, Xianzhi Liu ^1^*

**Supplementary Figure S1**

(A-B) Glioma cell lines, including C6 and LN18, were treated with varying concentrations of sorafenib for 24 h or 48 h. Cell viability was determined by MTT assay, and the results showed that sorafenib significantly decreased the viability of glioma cells in a time- and dose-dependent manner. Data are presented as the mean ± SD (n = 3).

**Supplementary Figure S2**

(A-B) Glioma cell lines, including C6 and LN18, were treated with varying concentrations of temozolomide (abbreviated as TMZ) for 24 h or 48 h. MTT assay showed that TMZ significantly decreased the viabilities of glioma cells in a time- and dose-dependent manner. (C) U251 and SHG-44 cells were treated with 2 μM sorafenib and 100 μM TMZ for 24 h. MTT assay showed a synergistic effect of the combination of sorafenib and TMZ in U251 and SHG-44 glioma cells for 24 h (*P < 0.05 and **P < 0.01 versus control group).

**Supplementary Figure S3**

(A) Quantitative result of Western blot assay demonstrated that the combination of sorafenib and TMZ upregulated the expression of Bax and Cyt c and downregulated the level of Bcl-2 (*P < 0.05 and **P < 0.01 compared to the control group). (B) Quantitative analysis of cytoplasmic and nuclear AIF protein in U251 cells for 24 h. The results showed that the combination of sorafenib and TMZ increased the nuclear aggregation of AIF (**P < 0.01 compared to the control group).

**Supplementary Figure S4**

AIF was knocked down in U251 and SHG-44 cells using small interfering RNA (siRNA). (A-B) After AIF was knocked down, quantitative analysis of Western blot assays showed that AIF siRNA downregulated the cytoplasmic and nuclear levels of AIF in U251 and SHG-44 cells. (C) After the same treatment, representative images with an optical microscope showed that siAIF mitigated the combination of sorafenib and TMZ-induced morphologic changes in U251 cells. (D) Quantitative analysis of Hoechst 33342 staining showed that siAIF mitigated the combination of sorafenib and TMZ-induced apoptotic changes to chromatin (**P < 0.01 compared to the control group).

**Supplementary Figure S5**

(A) Quantitative analysis of Western blot assays showed the level of p-STAT3 protein was downregulated in U251 cells treated with the combination of sorafenib and TMZ for 24 h (*P < 0.05 and **P < 0.01 versus control group). (B-D) Quantitative analysis of Western blot assays revealed that WP-1006 accelerated the sorafenib and TMZ treatment-induced translocation of AIF from mitochondria to nuclei in U251 cells.

**Supplementary Figure S6**

(A) Quantitative analysis of Western blot assays showed WP-1006 enhanced sorafenib and TMZ treatment-induced autophagy of U251 cells (*P < 0.05 and **P < 0.01 compared to the control group). (B) Quantitative analysis of Western blot assays revealed that the combination of sorafenib and TMZ increased the expression of autophagy-related protein Beclin 1 and WP-1006 promoted this upregulation of Beclin 1 in U251 cells (*P < 0.05 and **P < 0.01 versus control group).

**Supplementary Figure S7**

(A) Quantitative analysis of Western blot assays showed that the combination of sorafenib and TMZ decreased the level of phosphorylated JAK2 in U251 cells (*P < 0.05 compared to the control group). (B) Quantitative analysis of Western blot assays revealed that AG490 decreased the levels of phosphorylated JAK2 and STAT3 in U251 cells induced by the combination of sorafenib and TMZ (*P < 0.05 and **P < 0.01 compared to the control group). (C) Quantitative analysis of Western blot assays revealed that showed that NAC reversed the phosphorylation of JAK2 and STAT3 inhibited by the combination of sorafenib and TMZ (*P < 0.05 and **P < 0.01 compared to the control group, ^#^P < 0.05 versus the combination of sorafenib and TMZ)

**Supplementary Figures 8-14**

Captions in images
