# Supplementary figures and images for: Oxidative Stress Activated by Sorafenib Alters the Temozolomide Sensitivity of Human Glioma Cells Through Autophagy and JAK2/STAT3-AIF Axis
Source: Front Cell Dev Biol. 2021 Jun 14;9:660005. doi: 10.3389/fcell.2021.660005 (PMC8282178; doi:10.3389/fcell.2021.660005)

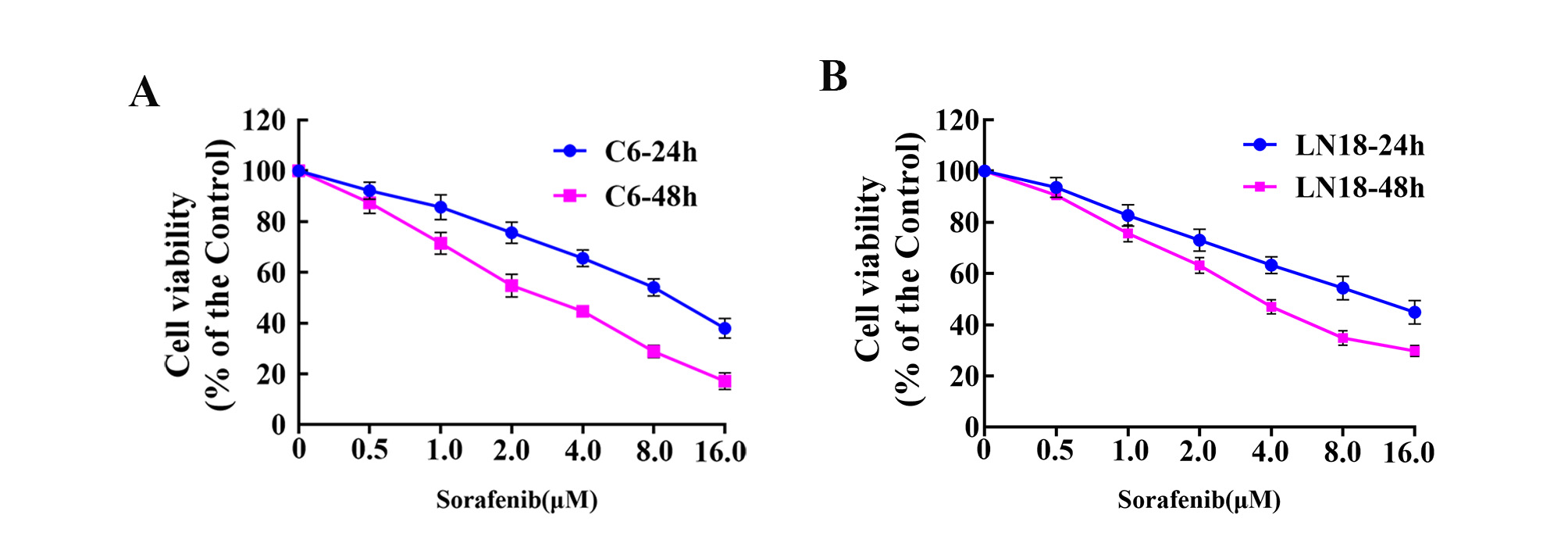

Supplement: Supplementary file 2 [file Image_1.jpeg]

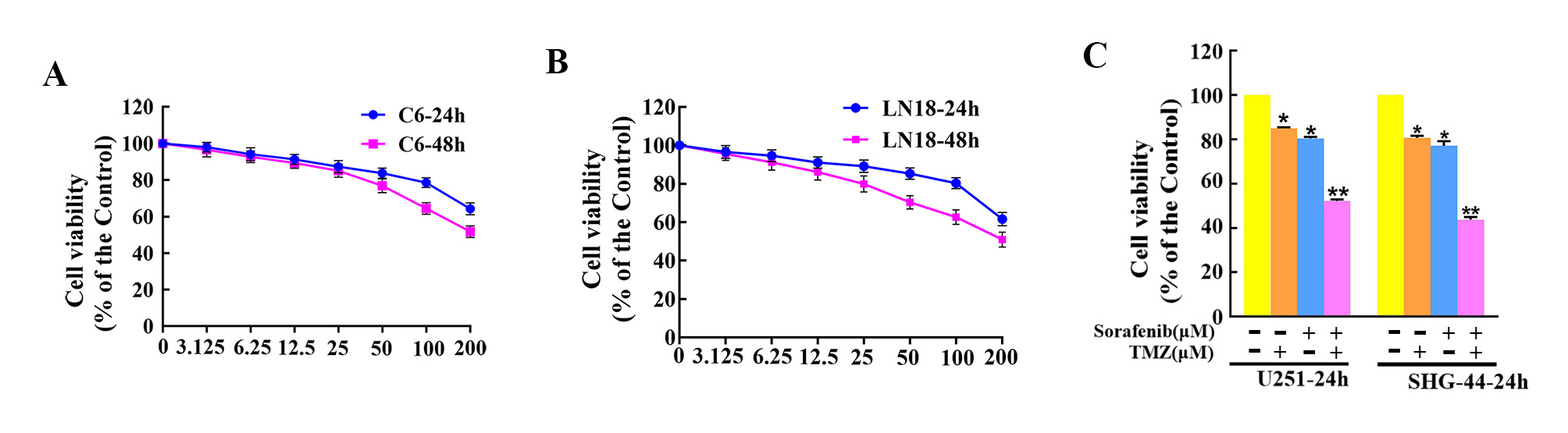

Supplement: Supplementary file 3 [file Image_2.jpeg]

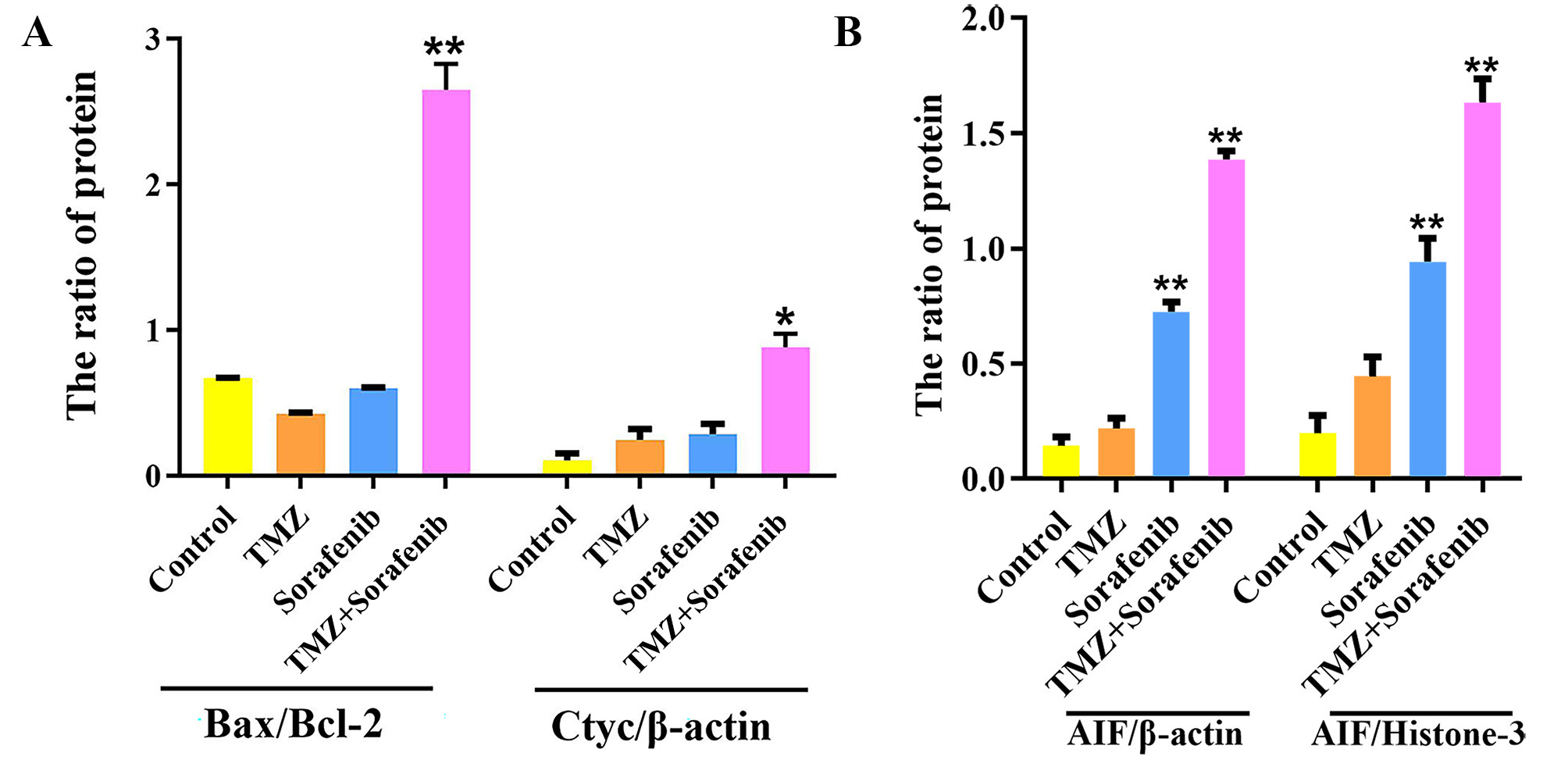

Supplement: Supplementary file 4 [file Image_3.jpg]

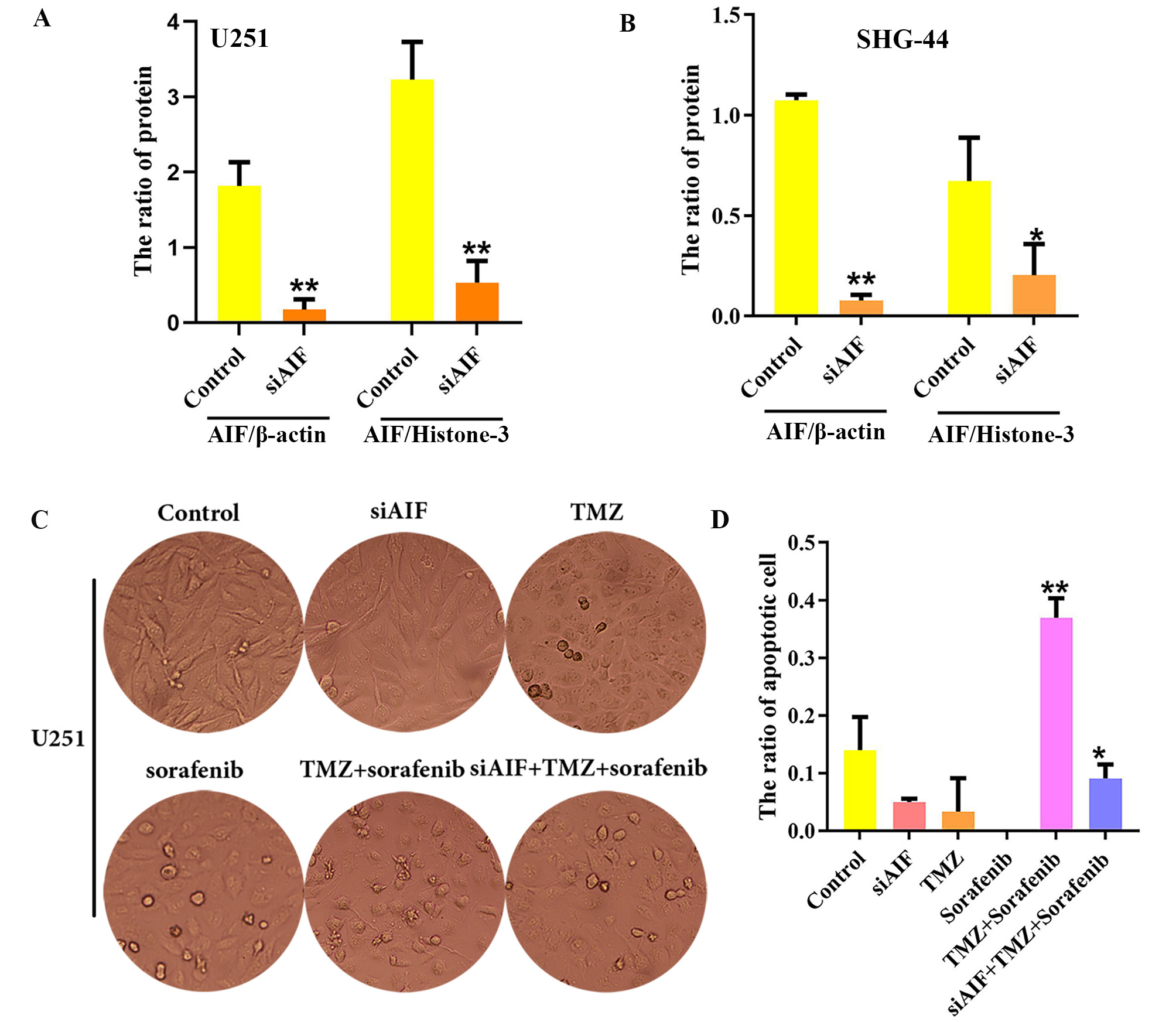

Supplement: Supplementary file 5 [file Image_4.jpeg]

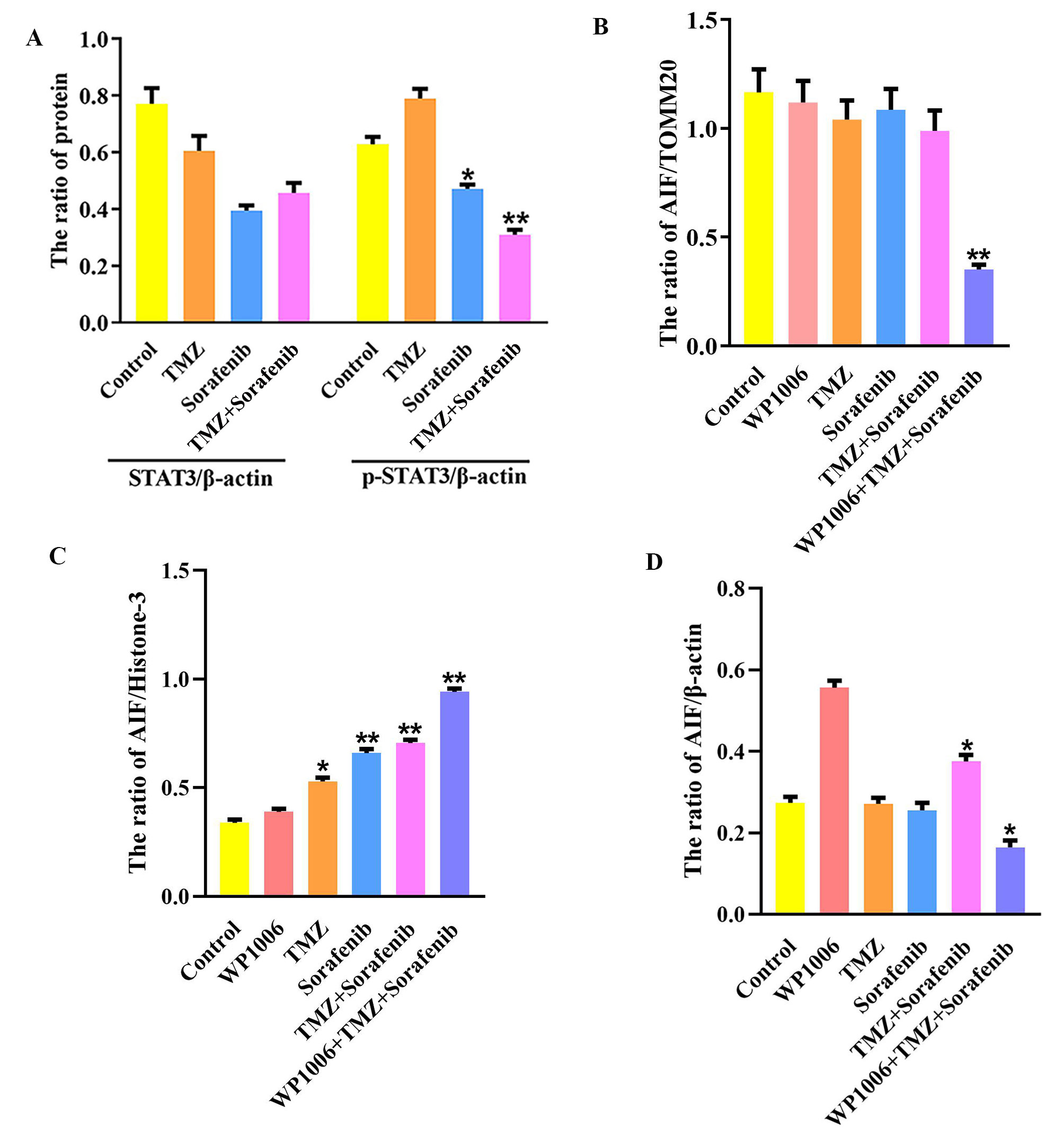

Supplement: Supplementary file 6 [file Image_5.jpg]

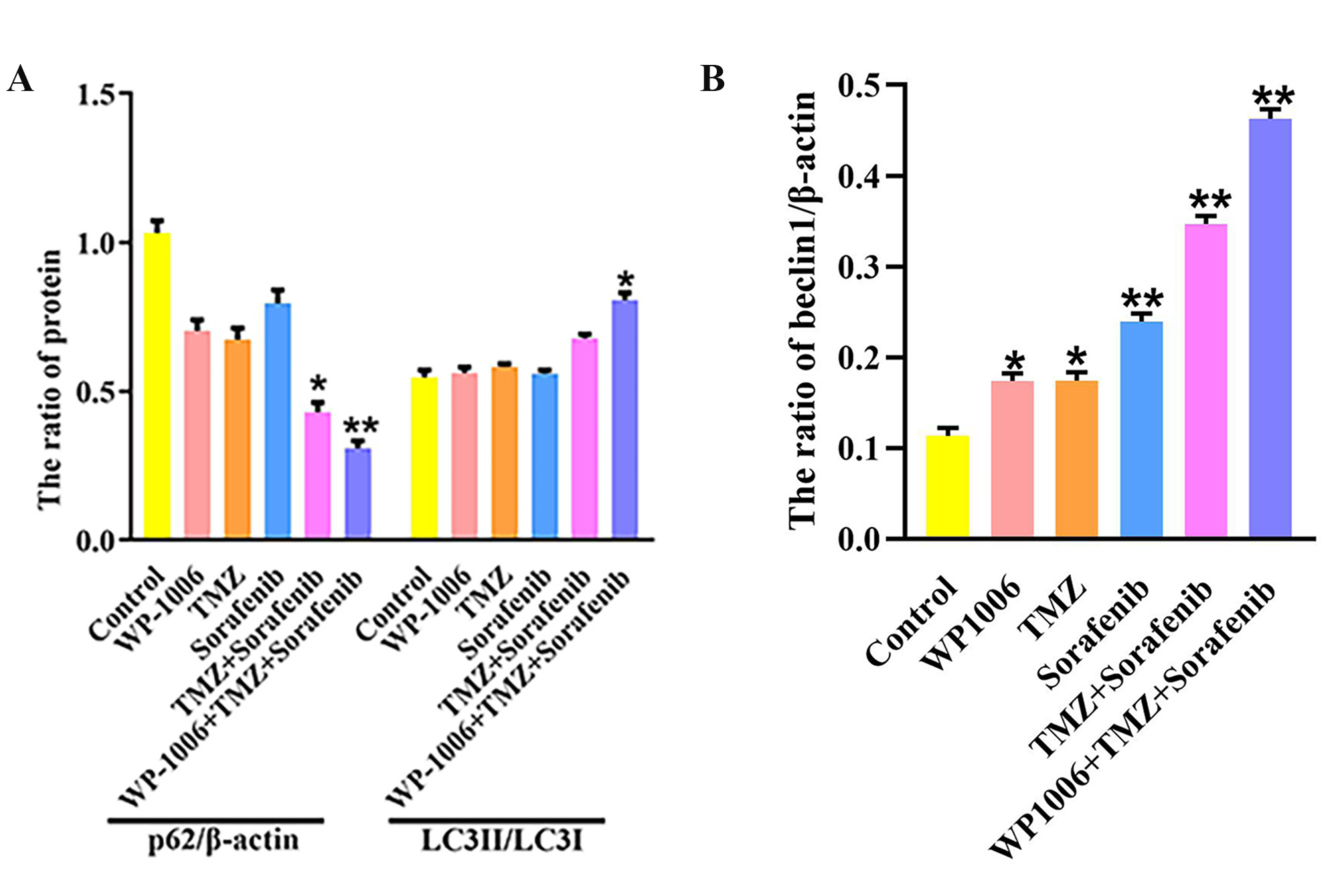

Supplement: Supplementary file 7 [file Image_6.jpeg]

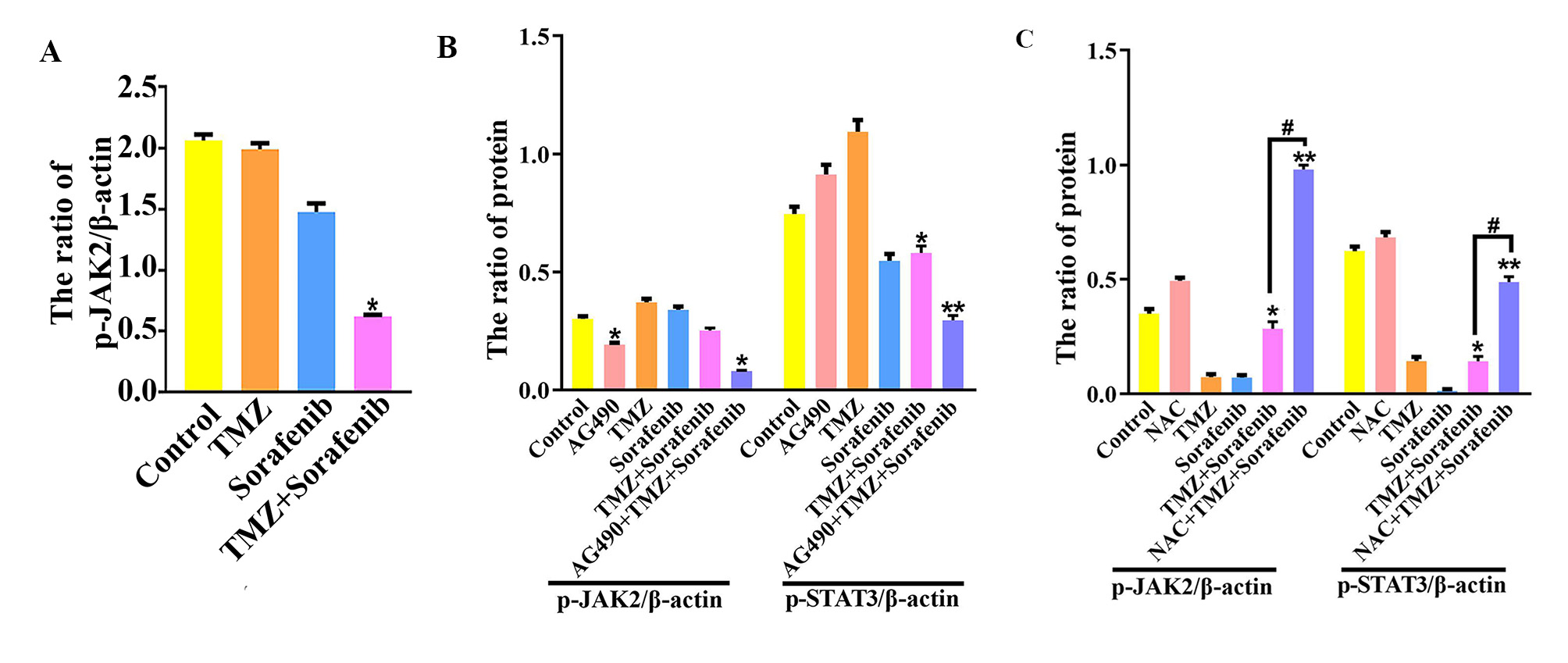

Supplement: Supplementary file 8 [file Image_7.jpeg]

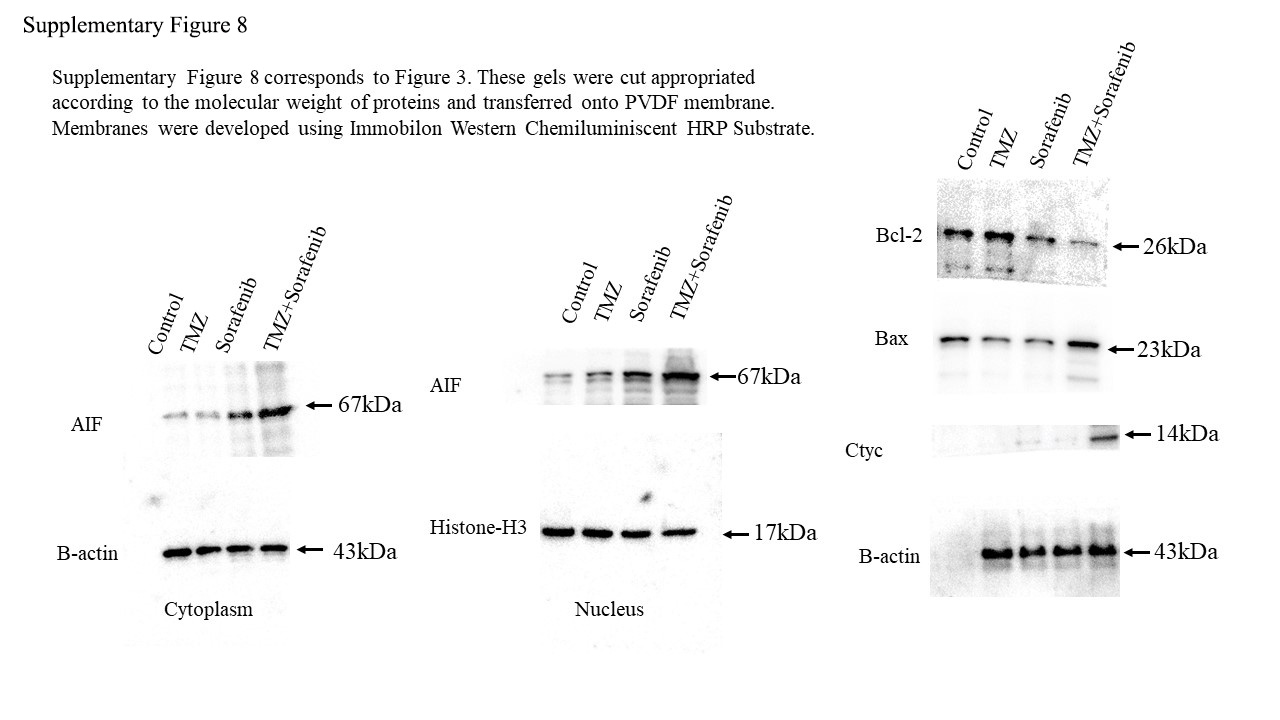

Supplement: Supplementary file 9 [file Image_8.jpg]

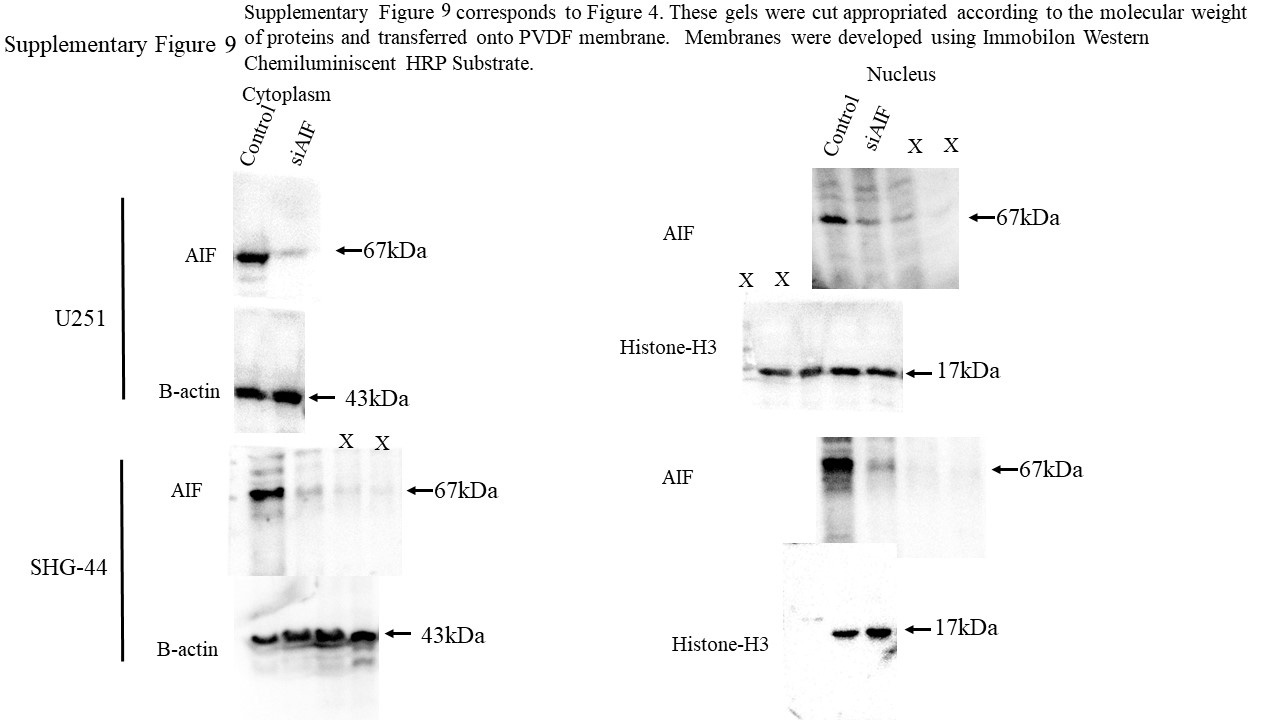

Supplement: Supplementary file 10 [file Image_9.jpg]

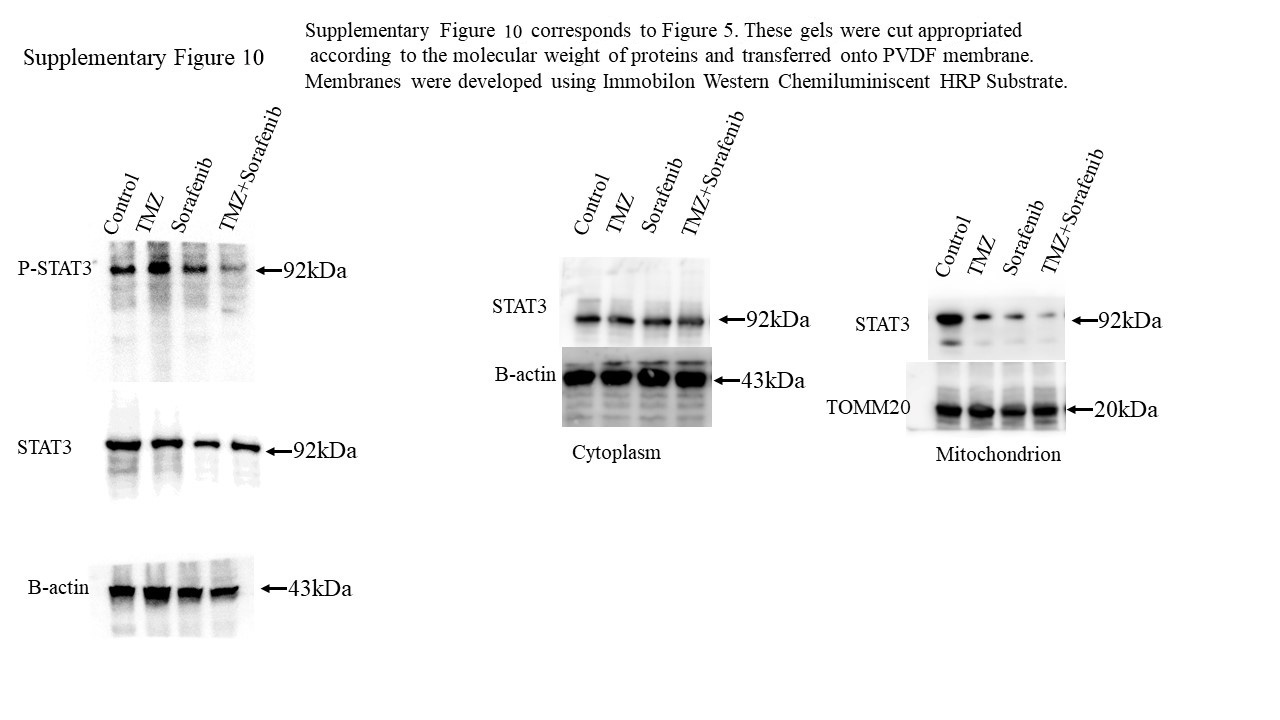

Supplement: Supplementary file 11 [file Image_10.jpg]

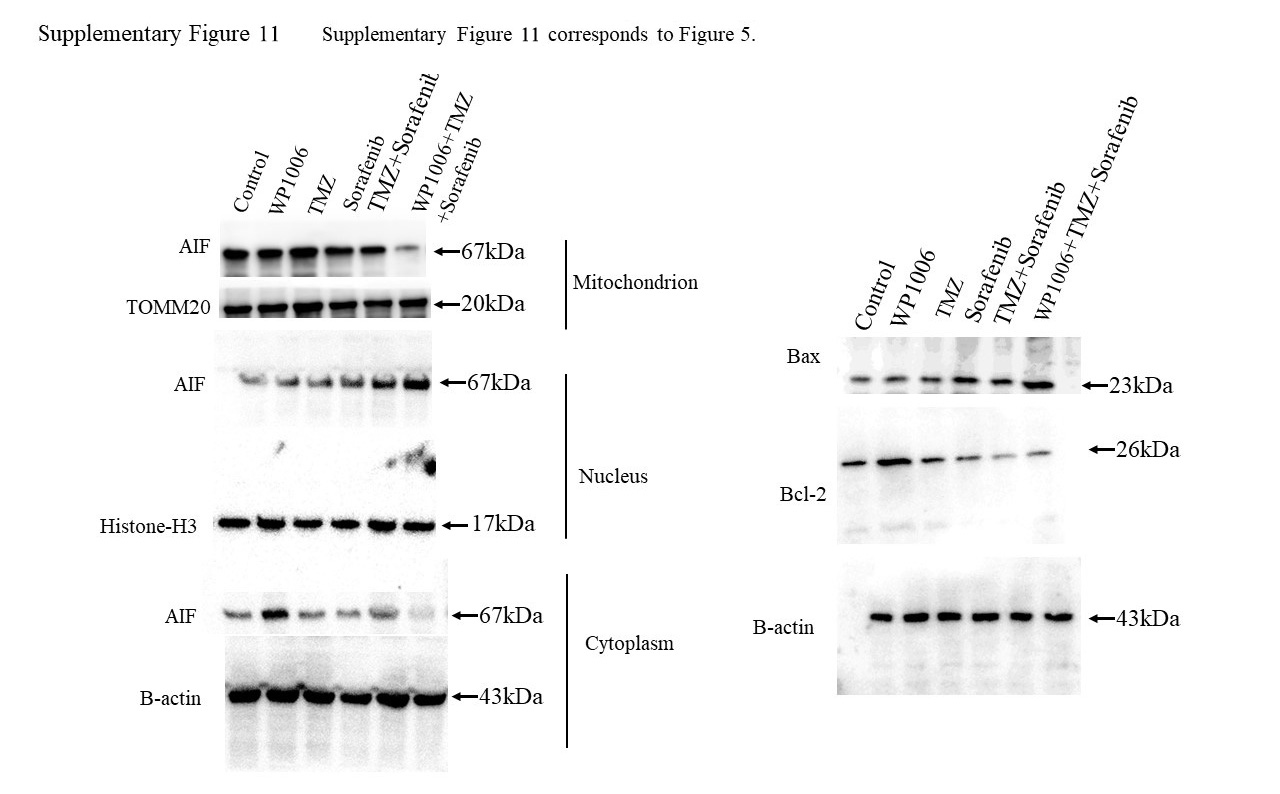

Supplement: Supplementary file 12 [file Image_11.jpg]

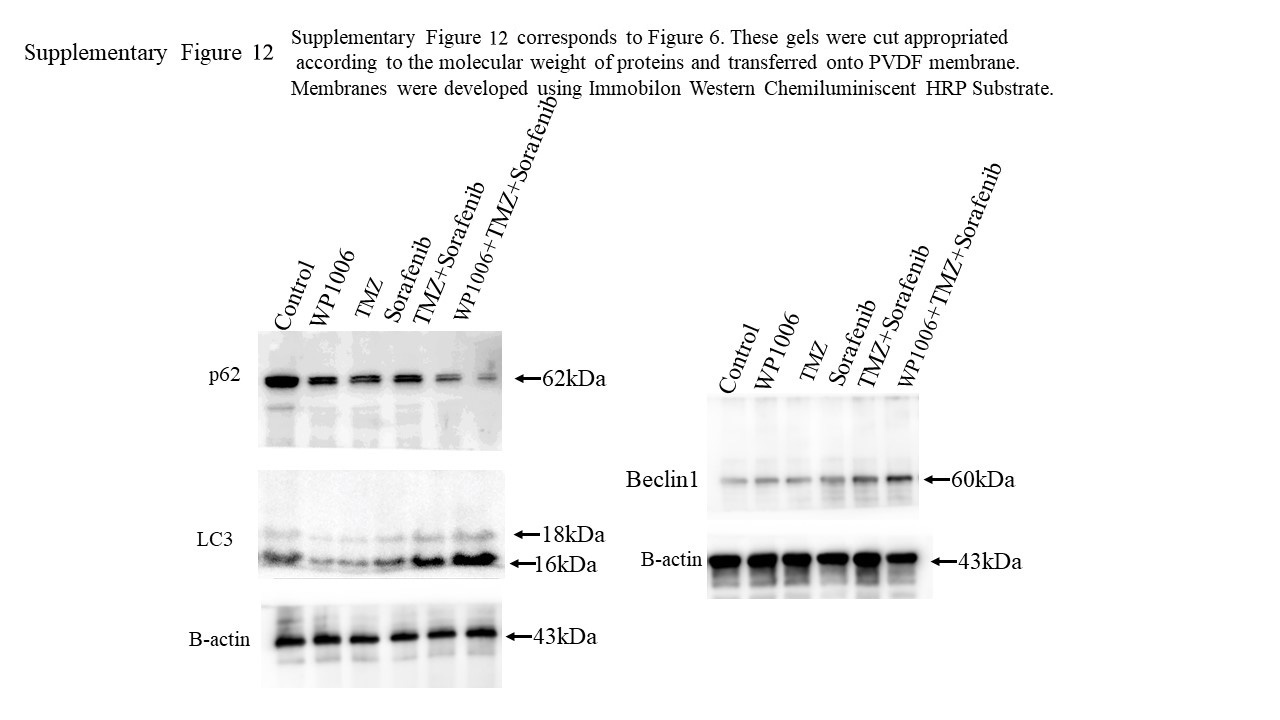

Supplement: Supplementary file 13 [file Image_12.jpg]

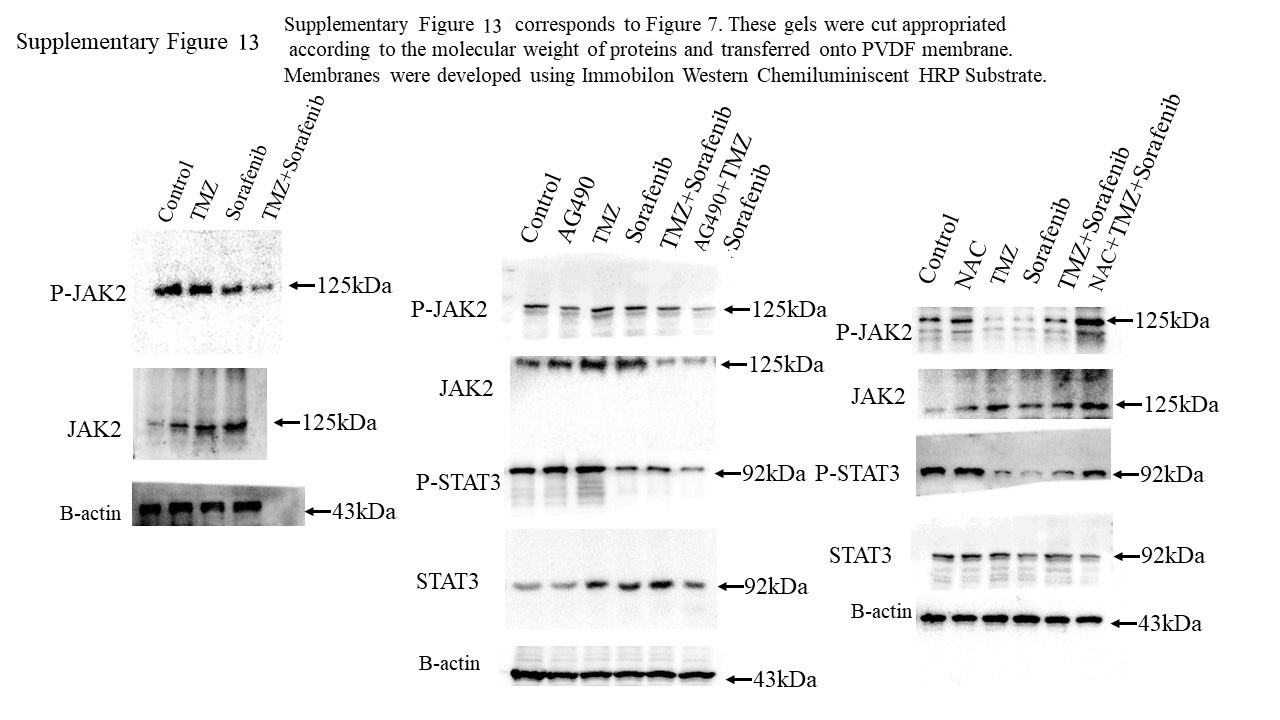

Supplement: Supplementary file 14 [file Image_13.jpg]

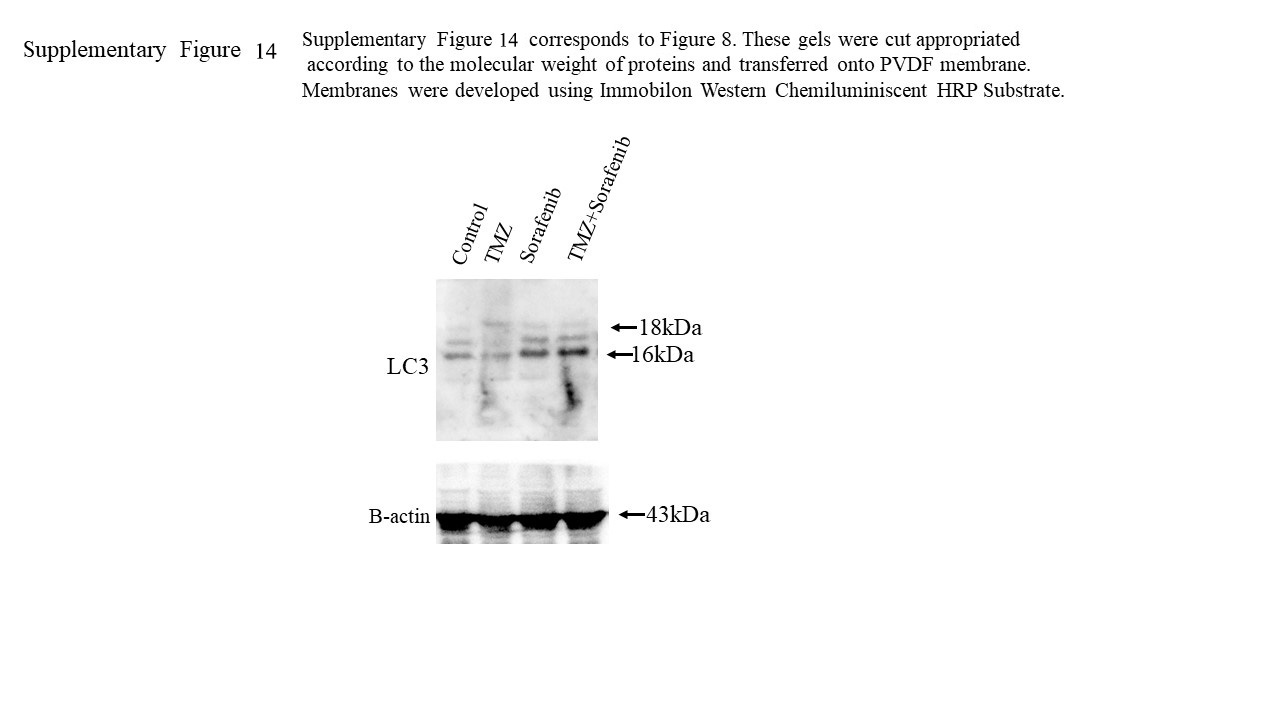

Supplement: Supplementary file 15 [file Image_14.jpg]
